# Phenanthrene: establishing lower and upper bounds to the binding energy of a very weakly bound anion†

**DOI:** 10.1039/d1cp04755h

**Published:** 2022-02-14

**Authors:** Elisabeth Gruber, Siegfried Kollotzek, Stefan Bergmeister, Fabio Zappa, Milan Ončák, Paul Scheier, Olof Echt

**Affiliations:** Institut für Ionenphysik und Angewandte Physik Universität Innsbruck Technikerstraße 25 6020 Innsbruck Austria Milan.Oncak@uibk.ac.at Paul.Scheier@uibk.ac.at; Department of Physics University of New Hampshire Durham NH 03824 USA olof.echt@unh.edu

## Abstract

Quite a few molecules do not form stable anions that survive the time needed for their detection; their electron affinities (EA) are either very small or negative. How does one measure the EA if the anion cannot be observed? Or, at least, can one establish lower and upper bounds to their EA? We propose two approaches that provide lower and upper bounds. We choose the phenanthrene (Ph) molecule whose EA is controversial. Through competition between helium evaporation and electron detachment in He_*n*_Ph^−^ clusters, formed in helium nanodroplets, we estimate the lower bound of the vertical detachment energy (VDE) of Ph^−^ as about −3 meV. In the second step, Ph is complexed with calcium whose electron affinity is just 24.55 meV. When CaPh^−^ ions are collided with a thermal gas of argon, one observes Ca^−^ product ions but no Ph^−^, suggesting that the EA of Ph is below that of Ca.

## Abstract 2

Collisional excitation of CaPh^−^, formed in helium nanodroplets, produces Ca^−^ but no phenanthrene anions. We conclude that the EA of Ph is below that of Ca, *i.e.* below 24.55 meV.

## Graphical abstract

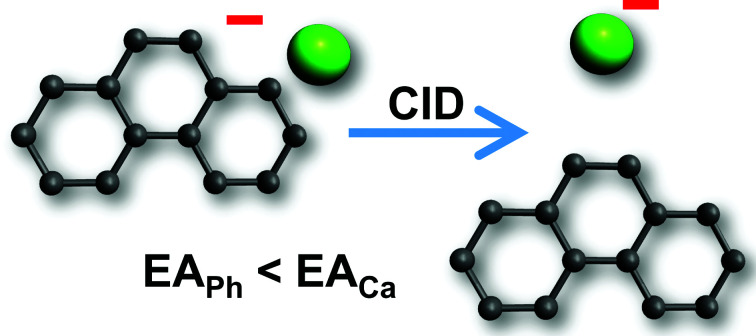

## Introduction

The electron affinities (EAs) of molecules are of interest not only when negative ions are encountered in the gas phase but also in condensed-matter chemistry; electron-transfer reactions play a role in organic, biological, and catalytic processes.^1^ However, many molecules have vanishingly small adiabatic electron affinities; several common molecules such as N_2_, H_2_O or CO_2_ do not form stable anions at all.^2^ It is difficult to measure the EA of a molecule M that does not strongly bind an electron. A compilation of EAs determined by the photothreshold or photoelectron spectroscopy approach^2^ lists a total of 1101 atoms or molecules; only 13 of those have an EA below 100 meV, another 11 have an EA below 300 meV. If M does not form a long-lived anion, how does one measure its EA?

In many cases, the lifetime of a weakly bound anion M^−^ is too short for its observation because of thermally activated electron emission. An obvious remedy is to lower the temperature. In the extreme case, one may try to form the anion within a liquid helium nanodroplet (HND) whose temperature is 0.37 K.^3^ Excess helium may then be removed by collisions with a helium gas, until the bare M^−^ emerges.^4^ But what if it doesn’t? There is another trick that the experimenter has in her toolbox, namely complexing M^−^ with a ligand X. For example, clusters of CO_2_ or H_2_O form stable anions where the excess electron is bound due to long-range correlations with the electrons or, in the case of polar molecules, in the dipole field.^2,5–11^ The EA of a water cluster as small as the dimer equals 43 meV even though the water monomer does not bind an electron.^12^

We demonstrate the viability of this two-pronged approach (synthesizing anions XM^−^ in liquid HNDs) by studying complexes of phenanthrene (Ph, C_14_H_10_) with various ligands whose electron affinity is negative (He, H_2_, H_2_O). With the exception of work by Lee *et al.*^13,14^ which will be discussed further below, Ph^−^ has so far escaped detection.^15–18^ Contradictory results have been reported for its electron affinity, namely ≈300 meV,^19–21^ 120 meV,^13^ and −10 ± 40 meV.^18^ The large (300 meV) values were obtained by the electron capture detection method whose reliability has been questioned;^18,22–24^ the 120 meV and −10 meV values are based on photoelectron (PE) measurements.^13,18^ A benchmark theoretical study of various polycyclic aromatic hydrocarbons places the EA of Ph at −80 meV.^24^ Several other theoretical studies agree that the EA is either very small, or negative.^18,25^

In the present work anions are formed by attaching electrons to helium nanodroplets (HNDs) which are subsequently passed through a pickup cell filled with a low-density vapor of phenanthrene, leading to large He_*n*_Ph^−^. Excess helium is then gently stripped from the doped, charged HNDs by multiple collisions with low-density helium gas until the emerging anions contain just a few helium atoms. Remarkably, we observe He_*n*_Ph^−^, *n* > 0, but no bare Ph^−^. The same is true if mass-selected He_*n*_Ph^−^, He_*n*_H_2_Ph^−^ or other small anionic complexes are collided with argon atoms. All possible anionic fragments of the precursor anion are detected, but bare Ph^−^ is not.

A density functional theory (DFT) study of various neutral and negatively charged complexes of Ph shows that helium increases the EA by just a few meV. If such a small increase is sufficient to drastically increase the lifetime of the anion, then its EA must be very small.

A more accurate upper bound to the EA is obtained by synthesizing He_*n*_CaPh^−^. The EA of Ca is just 24.55 meV.^26^ Upon collision with argon atoms, He_*n*_CaPh^−^ will shed its helium atoms and, eventually, dissociate into Ca^−^ + Ph rather than into Ca + Ph^−^. We conclude that the EA of Ph is less than 24.55 meV. Our approach is a variant of the well-established technique to bracket electron affinities by charge exchange reactions. If thermal collisions between M^−^ and X produce predominantly M + X^−^ then the EA of X is larger than that of M, because the branching ratio between two competing reaction channels in an activated system changes exponentially with the difference in the activation energies.^27^ The groups of Kebarle, Brauman, and Cooks, to name just a few, have made extensive use of this kinetic method to bracket EAs.^20,28–30^ Instead of studying the charge-exchange reaction (or its absence) between Ca^−^ and Ph, we study the half-reaction of CaPh^−^.

## Experimental details

Neutral HNDs are grown by supersonic expansion of helium through a nozzle (diameter 5 μm, temperature 8 K, stagnation pressure 25 bar) into ultra-high vacuum. The expanding beam is skimmed and ionized by electron attachment (energy 25 eV, current 330 μA). The resulting anions are weakly accelerated into an electrostatic hemispherical deflector set to transmit HNDs with a size-to-charge ratio *N*/*z* ≈ 3.5 × 10^6^, below the critical size for doubly charged HND anions.^31^ The charged HNDs pass through a pickup cell into which phenanthrene (Sigma Aldrich, 99.5%) is vaporized from an external oven kept at 50 °C, and an “evaporation cell” that contains helium at low, variable pressure *P*_evap_. Multiple collisions will lead to partial or complete evaporation of helium from the doped HND. The helium pressure is tuned until negatively charged complexes of Ph and He emerge that contain just a few helium atoms. The ions are guided by a radio-frequency field into the extraction region of a time-of-flight mass spectrometer (TOFMS) equipped with a reflectron in V-configuration. The products of collision-induced dissociation of mass-selected ions are determined by first passing the ions that emerge from the evaporation cell through a quadrupole mass filter and then through a cell filled with argon gas at ambient temperature.

Negatively charged complexes containing Ph and Ca are formed by evaporating Ca and Ph in two separate pickup cells. Other ligands (H_2_O, H_2_) result from collisions of the HNDs with residual gas, or ion–molecule reactions. Further details are described elsewhere.^3,32,33^

## Computational details

There is a vast body of theoretical work that has been done to estimate the stability of very weakly bound, or even unbound, negative anions.^2,18,22–25,34,35^ We have performed quantum chemical calculations of various Ph complexes using DFT along with the D3 dispersion correction as suggested by Grimme *et al.*^36^ Our calculations cannot asses electron affinities quantitatively, among other factors due to their high sensitivity to the zero-point energy correction as already discussed elsewhere.^18^ However, results obtained using the *ω*B97XD functional seem to be in reasonable agreement with available experiments. For example, calculated vertical detachment energies (VDE) of (H_2_O)_*n*_Ph^−^, *n* = 1–3, are overestimated on average by 65 and 170 meV at the *ω*B97XD/aug-cc-pVDZ and B3LYP-D3/aug-cc-pVDZ levels, respectively, compared to the experiment,^18^ see the ESI† (Fig. S6). The VDE of Ph_2_^−^ is calculated as 249 and 527 meV at the same levels of theory, the experimental value being 270 meV.^13^ All used DFT functionals predict similar shifts in electron affinities of Ph when complexed with He, H_2_ and H_2_O. Only *ω*B97XD and B3LYP-D3 results are discussed below; benchmarking calculations can be found in the ESI† (Tables S1 and S2). Electron affinities include the zero-point correction, vertical detachment energies do not. Note that due to the system size, the zero-point correction is calculated within the harmonic approximation, which might lead to inaccuracies especially for neutral systems with attached helium atoms. Cluster structures were optimized using very tight convergence criteria; wave function stability was tested prior to every calculation. Complexes with more than one adsorbed atom or molecule are included in Fig. S5 and S6 (ESI†). All calculations were performed in the Gaussian software package.^37^

## Results and discussion

**Fig. 1:**
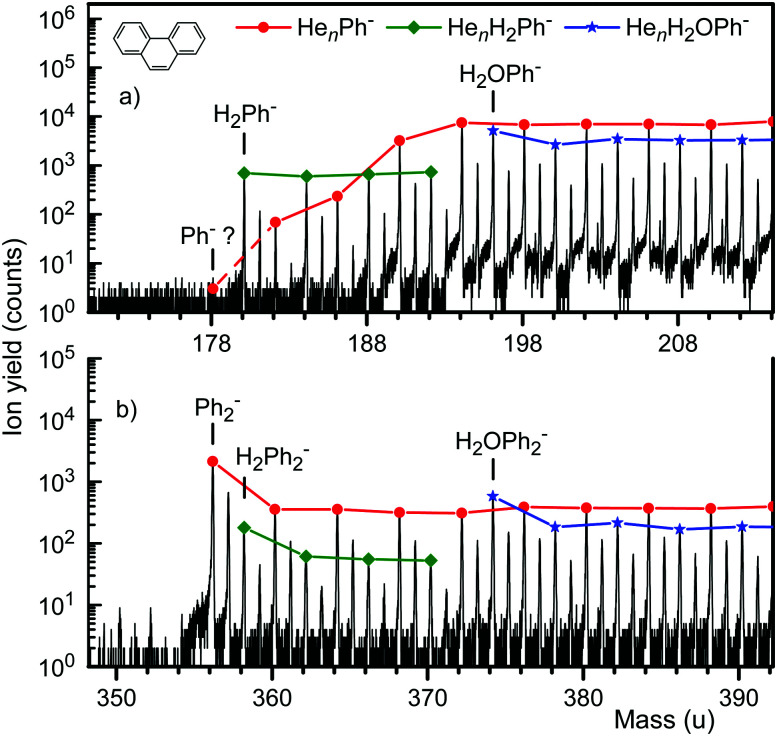
Two sections of a negative ion mass spectrum of helium nanodroplets (HND) doped with phenanthrene (Ph, C_14_H_10_). Anionic complexes of Ph with *n* > 0 helium atoms, or with H_2_ or H_2_O plus *n* ≥ 0 helium atoms are observed in panel a, but bare Ph^−^ is noticeably absent. Panel b shows the equivalent mass range for ions containing two Ph molecules. Ph_2_^−^ forms a prominent mass peak.

A mass spectrum of HNDs doped with Ph is displayed in Fig. 1. Three distinct homologous ion series appear in Fig. 1a: He_*n*_Ph^−^, He_*n*_H_2_Ph^−^, and He_*n*_H_2_OPh^−^. Mass peaks due to ions that contain the main isotopes of each element (^1^H, ^4^He, ^12^C, and ^16^O) are marked by symbols; connecting lines are drawn to guide the eye. The first member of each series (*i.e.* ions containing no helium, *n* = 0) is labeled.^38^

The presence of ions containing a water impurity is unavoidable when working with very large HNDs. Tschurl *et al.* have reported PE spectra of (H_2_O)_*n*_Ph^−^ (*n* = 1, 2, 3, *n* > 0); the ions were prepared by seeding an expanding nitrogen gas with phenanthrene and water.^18^ They obtained a VDE of 270 ± 20 meV for H_2_OPh^−^; bare Ph^−^ could not be observed.

Two features in Fig. 1a are striking: The presence of HePh^−^ and H_2_Ph^−^, and the absence of Ph^−^ which cannot be positively identified; its yield is less than 3% relative to that of HePh^−^, and less than 0.3% relative to H_2_Ph^−^. HePh^−^ and H_2_Ph^−^ are very weakly bound (see below). The temperatures of the observed anions HePh^−^ and H_2_Ph^−^ must be correspondingly low,^39^ hence the absence of Ph^−^ suggests that the excess electron is very weakly bound.

Fig. 1b displays another section of the mass spectrum, revealing the same homologous ion series as in panel a but involving Ph_2_ rather than Ph. Bare Ph_2_^−^ forms a strong mass peak. Homologous ion series based on Ph_3_^−^ (see the ESI†) and larger Ph_*m*_^−^ cluster ions are seen as well. The observation of intense Ph_*m*_^−^ signals for *m* > 1 agrees with previous work by Lee *et al.* who formed Ph_*m*_^−^ by expansion of Ph vapor seeded in argon gas; free electrons were attached to Ph clusters in the expansion region.^13,14^

The ions that appear in Fig. 1 result from multiple collisions of large, doped, negatively charged HNDs with helium atoms at thermal energies. Each collision will transfer, on average, 0.05 eV to the HND, about 80 times the evaporation energy of bulk helium. The spectrum does not convey any information about the immediate precursors of the observed ions or, turned around, the dissociation channels of a given ion. This information can be garnered from collision-induced-dissociation (CID) spectra, which were recorded by passing the ions that emerge from the evaporation cell through a quadrupole mass filter. The selected precursor ions are accelerated and sent into a cell where they collide with a thermal gas of argon; product ions are then analyzed in the TOFMS.^32,33,40^

**Fig. 2:**
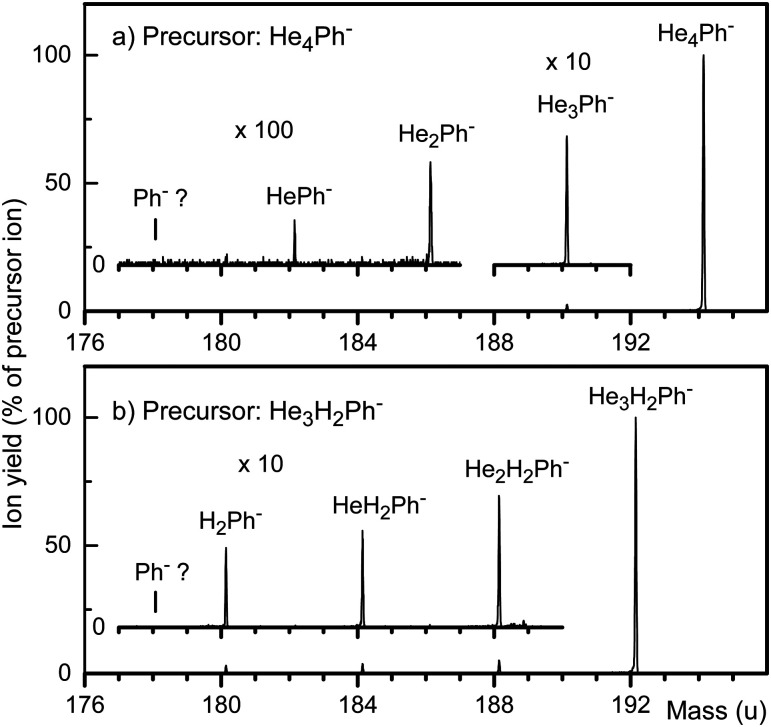
Mass spectra of ions produced by collisions of precursor ions He_4_Ph^−^ and He_3_H_2_Ph^−^ (panels a and b, respectively) with argon atoms at 2 eV ion energy (in the lab system) and an argon gas pressure of 0.8 mPa. Various fragment ions are observed, but no bare Ph^−^.

Two CID spectra are presented in Fig. 2. The relative yield of He_*n*_Ph^−^ fragments from the precursor ion He_4_Ph^−^ (panel a) decreases rapidly from 2.5% for He_3_Ph^−^ to 0.09% for HePh^−^. Ph^−^ cannot be identified; its relative yield is less than 0.005% of the precursor, or 5% of HePh^−^. Thus, when He_4_Ph^−^ is excited by collisions, it may shed one, two or three atoms without losing its electron,^41^ but the electron will detach upon loss of the fourth and last helium atom.

Fig. 2b displays a CID spectrum of He_3_H_2_Ph^−^. The relative yield of product ions due to loss of one, two, or three He equals a few percent but no bare Ph^−^ is detected. Its relative yield is less than 0.002% of the precursor, or 0.06% of H_2_Ph^−^. The preference for He loss rather than H_2_ loss is not surprising, given that the polarizability of H_2_ is nearly four times larger than that of He.

The data in Fig. 2a and b reveal a striking difference between the ion series He_*n*_Ph^−^ and He_*n*_H_2_Ph^−^; the yield of the former increases rapidly with size *n* while that of the latter remains constant. The same trends are apparent in the mass spectrum in Fig. 1a (note the logarithmic scale). We tentatively attribute the rapid increase of the He_*n*_Ph^−^ yield to its very low stability for small values of *n*. Even a slight increase in its stability with increasing *n*, as discussed further below, will then greatly extend its lifetime. He_*n*_H_2_Ph^−^, on the other hand, is already quite stable even if *n* = 0.

The CID spectra of (H_2_O)_*n*_Ph^−^ and Ph_*m*_^−^ are presented in the ESI.† These anions shed their ligands (H_2_O and Ph, respectively) upon collision-induced dissociation, but bare Ph^−^ is not produced. To summarize, any of the ligands explored so far (He, H_2_, H_2_O, Ph) will stabilize Ph^−^, but the EA of bare Ph is too small (or perhaps even negative) for the detection of its anion.

**Fig. 3:**
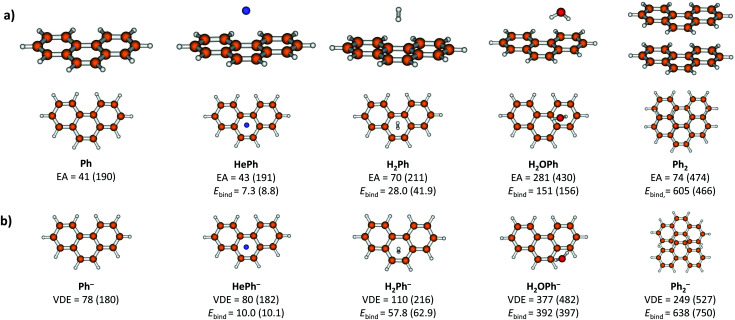
Structures of Ph, HePh, H_2_Ph, H_2_OPh and Ph_2_ shown in side and top views (a) and their anionic counterparts shown in top view (b) along with adiabatic electron affinities (EA), vertical detachment energies (VDE) and binding energies (*E*_bind_), all in meV. Energies are given as calculated at the *ω*B97XD/aug-cc-pVDZ level; B3LYP-D3/aug-cc-pVDZ results are shown in parenthesis. Structures optimized at the B3LYP-D3/aug-cc-pVDZ level are displayed. Note that the VDE values are not zero-point corrected, leading to VDE < EA for Ph and HePh. The considerable difference in EA(Ph_2_) for the two functionals can be traced to different structures of Ph_2_^−^, see Fig. S7 (ESI†).

Calculated complexes of Ph with He, H_2_, H_2_O and Ph are shown in Fig. 3, along with binding energies, vertical detachment energies and electron affinities. The binding energies of HePh and HePh^−^ were evaluated as 7.3 and 10.0 meV, respectively, at the *ω*B97XD/aug-cc-pVDZ level (Fig. 3). The stronger interaction with He in the anion compared to the neutral molecule leads to an increased electron affinity of HePh compared to Ph; the difference, however, is of the order of meV. For He_*n*_Ph^−^, *n* = 1–3, our calculations show that each helium atom increases both electron affinity and vertical detachment energy by about 1–3 meV (Fig. S5, ESI†), in agreement with the trend observed in the experiment.

Calculations on HePh^−^ give us the possibility to estimate the lower bound of the phenanthrene electron affinity. As the HePh^−^ ion is observed in the experiment, VDE(HePh^−^) should be >0 meV. The computed VDE of Ph^−^ is 2–3 meV lower than that of HePh^−^, hence VDE(Ph^−^) >≈ −3 meV. On the other hand, the upper bound of the VDE should not be much higher than several meV as the Ph^−^ ion itself is not observed in the mass spectrum. The electron affinity must be lower than the VDE; if zero-point effects are neglected, the difference between EA and VDE in phenanthrene is calculated as 191 (150) meV employing the *ω*B97XD (B3LYP) functional, in reasonable agreement with a previous calculation of 153 meV,^18^ see Table S3 (ESI†). This suggests that the He_*n*_Ph^−^ ions observed in the experiment are metastable for small *n*, and the method could be used for preparing metastable anionic species for further spectroscopic studies.

Similarly, complexation of Ph with H_2_, H_2_O and Ph leads to an increase in electron affinity due to a stronger interaction in the anionic molecule compared to the neutral one. In (H_2_)_*n*_Ph^−^ and (H_2_O)_*n*_Ph^−^ complexes, each H_2_ and H_2_O increases the electron affinity by about 15 to 30 and 190 to 240 meV, respectively, for *n* = 1–3 (see Fig. S5 and S6, ESI†). Finally, the vertical detachment energy of Ph_2_^−^ was calculated to lie about 200–350 meV above that of Ph (Fig. 3), rationalizing observation of this ion in the experiment.

We can also establish an experimental upper bound of the electron affinity by investigating negatively charged complexes of Ph with Ca. The EA of Ca, 24.55 meV, is smaller than that of any other atom that forms a stable anion.^26^ If CaPh^−^ is mildly excited in low-energy collisions, what are the products?

**Fig. 4:**
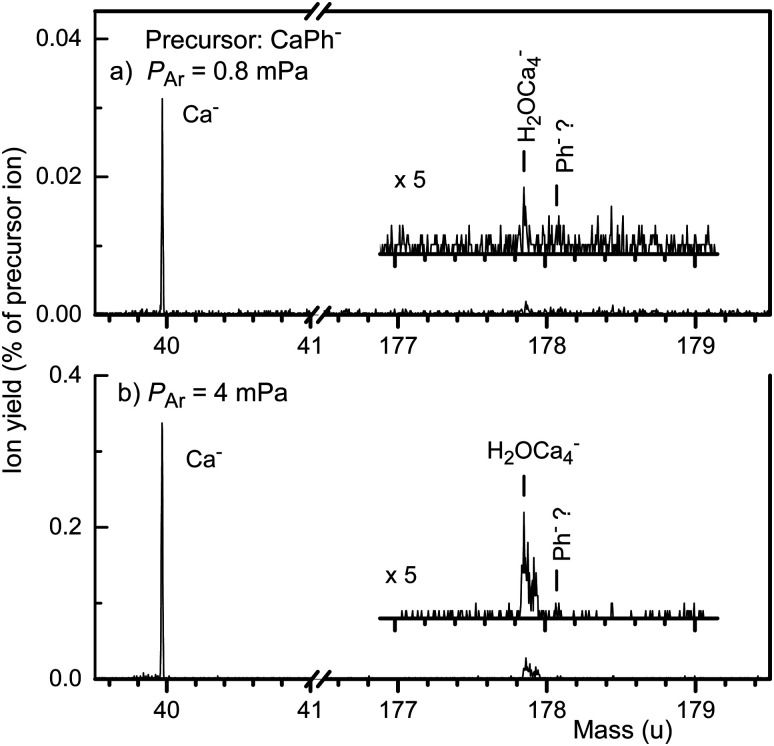
Mass spectra of ions produced by collisions of CaPh^−^ with argon atoms at 5 eV ion energy (in the lab system) at two different argon pressures (panels a and b). The main product ion is Ca^−^. A contamination of the parent ion mass peak at 218 u by H_2_OCa_5_^−^ gives rise to H_2_OCa_4_^−^ product ions. Ph^−^ is not positively identified.

The main isotope of Ca is ^40^Ca (mass 39.963 u, abundance 96.941%). When Ph and Ca are co-vaporized in the pickup cell, the mass peak at nominally 218 u in the negative ion mass spectrum contains contributions from ^40^CaPh^−^, but H_2_O^40^Ca_5_^−^ and He_10_Ph^−^ contribute as well. Their mass peaks are resolved in the TOF spectrum but the quadrupole mass filter that selects ions for the CID measurements cannot separate those precursor ions. Sections of a CID spectrum of mass 218 ions are displayed in Fig. 4; complete spectra are presented in the ESI.†^40^Ca^−^ forms, by far, the most prominent product ion peak; its relative yield increases from 0.03% to 0.3% as the pressure of the argon collision gas is raised from 0.8 mPa to 4 mPa (panels a and b, respectively). A much weaker signal appears at 178 u which, however, is due to Ca loss from H_2_OCa_5_^−^ rather than Ca loss from CaPh^−^. Once again, Ph^−^ cannot be positively identified.

The full CID mass spectrum of mass 218 ions (see ESI†) reveals a few other mass peaks that are due to He loss from He_10_Ph^−^ or loss of one or more Ca atoms from H_2_OCa_5_^−^. A critical reader might argue that Ca^−^ product ions originate from H_2_OCa_5_^−^ rather than from CaPh^−^. This is, however, easily ruled out by turning off the Ph source: As shown in the ESI,† the Ca^−^ signal disappears to <0.0001% of the precursor ion while ions due to loss of H_2_O, H_2_, and one or two Ca atoms from H_2_OCa_5_^−^ persist at a few %.

So far, we have not considered another possible reaction channel, namely electron detachment. Our setup is blind to this channel; we cannot detect neutral products nor free electrons. Competition between electron detachment and dissociation occurs, for example, for (H_2_O)_*n*_^−^.^8^ The relative yield of the competing channels does not only depend on the activation energies but also on the excess energy available.^42^ Still, electron detachment would merely reduce the anion yield; it would not affect the competition between formation of Ca^−^ and Ph^−^.

Hence the main result is that Ca^−^ is the only fragment ion produced by collisional excitation of CaPh^−^. No Ph^−^ ions are detected. We conclude that the EA of Ph is well below that of Ca (24.55 meV), and certainly not much larger. The conclusion is consistent with PE data by Tschurl *et al.*,^18^ but at variance with PE data by Lee *et al.*^13,14^ Lee *et al.* observed prominent mass peaks due to Ph_*m*_^−^ ions, *m* > 1, and a very weak mass peak near 178 u that they assigned to bare Ph^−^. They deduced a VDE of 120 meV from a PE spectrum of these ions. Their stated mass resolution, however, was just 1/200; the full width of mass peaks in their published data measures about 2.5 u. It is conceivable that the true mass of the observed anions differed slightly from 178 u. They used a primary electron beam of 400 eV; secondary electrons were assumed to attach to Ph to produce Ph^−^. Ph has a strong resonance at 7.7 eV for dissociative attachment to produce (Ph-H)^−^.^17^ Alternatively, the PE spectrum reported by Lee *et al.* might be due to H_2_Ph^−^ which contributes strongly to the negative ion mass spectrum as seen in Fig. 1. Its PE spectrum would probably resemble that of Ph_*m*_^−^, *m* > 1, except for a spectral blue shift. On the other hand, the measured blueshift^13,14^ is significantly larger than the one predicted by our calculations.

## Conclusion

To summarize, we have shown that long-lived He_*n*_Ph^−^ anions can be formed in HNDs and fragmented by low-energy collisions with Ar into He_*x*_Ph^−^ as small as *x* = 1. However, bare Ph^−^ cannot be observed although calculations show that a single helium atom increases the EA of Ph by just a few meV. If an increase in the EA by a few meV stabilizes the anion, its EA as well as its temperature must be very low. More quantitatively, we observe that HePh^−^ is stable while Ph^−^ is not, enabling us to estimate the lower bound of VDE(Ph^−^) as ≈−3 meV. At the same time, collisions of CaPh^−^ produce Ca^−^ but no Ph^−^, indicating that the EA of Ph is below that of Ca, *i.e.* below 24.55 meV. This work shows that very weakly bound, previously unobservable anions can be formed in HNDs; it outlines a method to determine bounds to their VDE and EA, and proposes VDE(Ph) > −3 meV and EA(Ph) < 24.55 meV.

## Conflicts of interest

The authors declare no conflict of interest.

## Supplementary Material

CP-024-D1CP04755H-s001
