# Supplementary material for: Phenanthrene: establishing lower and upper bounds to the binding energy of a very weakly bound anion
Source: Phys Chem Chem Phys. 2022 Feb 14;24(8):5138–43. doi: 10.1039/d1cp04755h (PMC8865840; doi:10.1039/d1cp04755h)
Supplement: CP-024-D1CP04755H-s001 [file CP-024-D1CP04755H-s001.pdf]

Electronic Supplementary Material (ESI) for Physical Chemistry Chemical Physics.
This journal is © the Owner Societies 2021

Electronic supplementary information (ESI)

Phenanthrene: Establishing lower and upper bounds to the binding energy of a very weakly bound anion

Elisabeth Gruber,[†a] Siegfried Kollotzek,[†a] Stefan Bergmeister, Fabio Zappa,[a] Milan Ončák,[*a] Paul Scheier,[*a] Olof Echt[*ab]

a Institut für Ionenphysik und Angewandte Physik, Universität Innsbruck, Technikerstraße 25, 6020 Innsbruck (Austria)
b Department of Physics, University of New Hampshire, Durham, NH 03824 (USA)
† Both authors contributed equally
* Corresponding Authors: Milan Ončák <Milan.Oncak@uibk.ac.at>; Paul Scheier <Paul.Scheier@uibk.ac.at>; Olof Echt < olof.echt@unh.edu>

- Fig. S1: Negative ion mass spectrum of HNDs doped with Ph.
- Fig. S2: CID spectrum of $(H_2O)_3Ph^-$ and Ph_2^- ions.
- Fig. S3: CID spectrum of mass 218 ions, including $CaPh^-$ (HNDs doped with Ca and Ph).
- Fig. S4: CID spectrum of mass 218 ions, excluding $CaPh^-$ (HNDs doped with Ca but no Ph).
- Fig. S5: Electron affinities and binding energies for phenanthrene complexed with He_n, n = 1-3, and $He.H_2$.
- Fig. S6: Electron affinities and binding energies for phenanthrene complexed with $(H_2)_n$ and $(H_2O)_n$, n = 1-3.
- Fig. S7: Optimized structure of Ph_2^-.
- Table S1: Method benchmark for electron affinities.
- Table S2: Method benchmark for vertical detachment energies.
- Table S3: The effect of zero point energies for neutral and anionic Ph and HePh.
- Cartesian coordinates of optimized structures

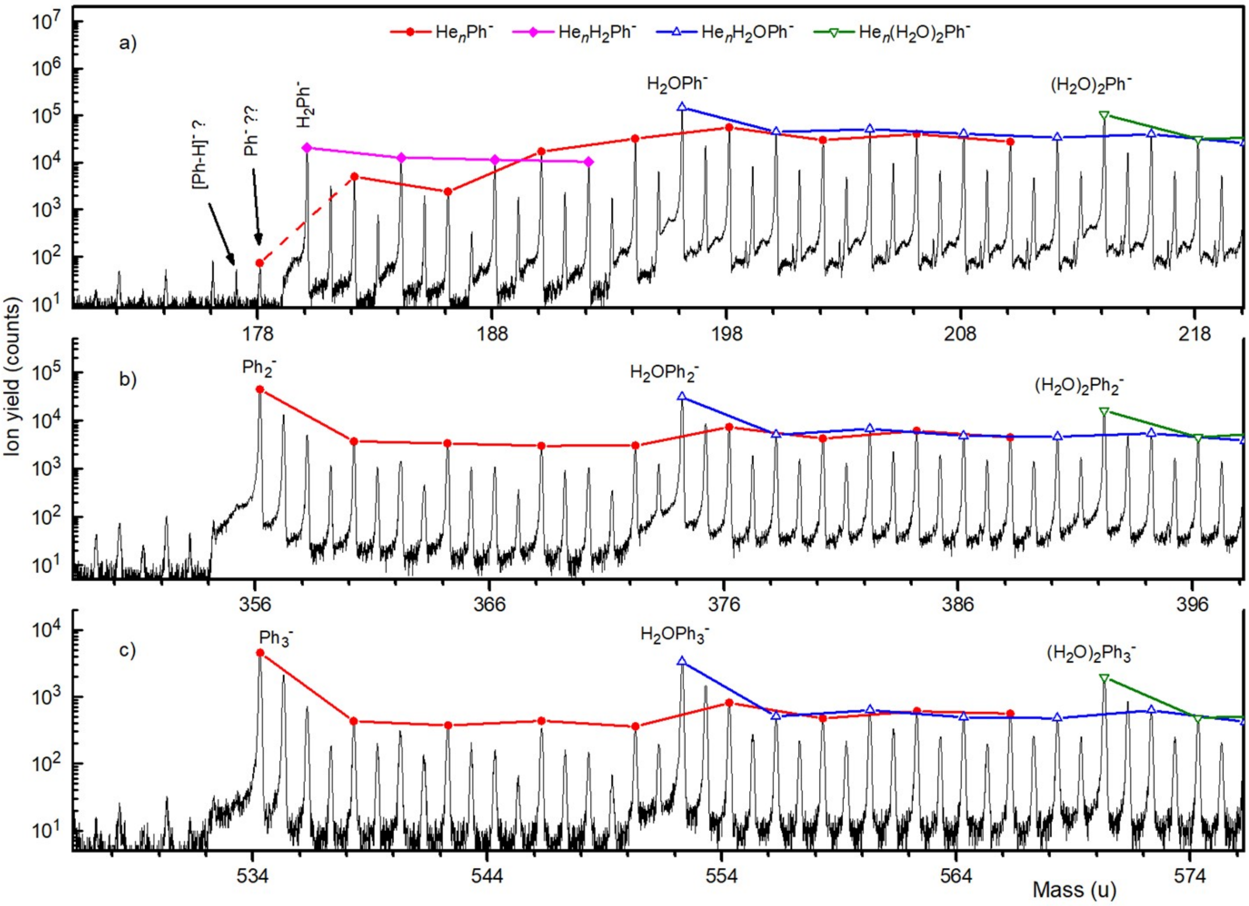

Fig. S1
Sections of a negative ion mass spectrum of HNDs doped with phenanthrene. Panels a, b, c show the regions where Ph^-, Ph_2^- and Ph_3^- complexed with a few He atoms appear. The *x*-axis is chosen such that members of the homologous $He_nPh_m^-$ ion series (m = 1, 2, 3) with identical n are horizontally aligned. Ph_2^- and Ph_3^- are observed, but Ph^- is not.

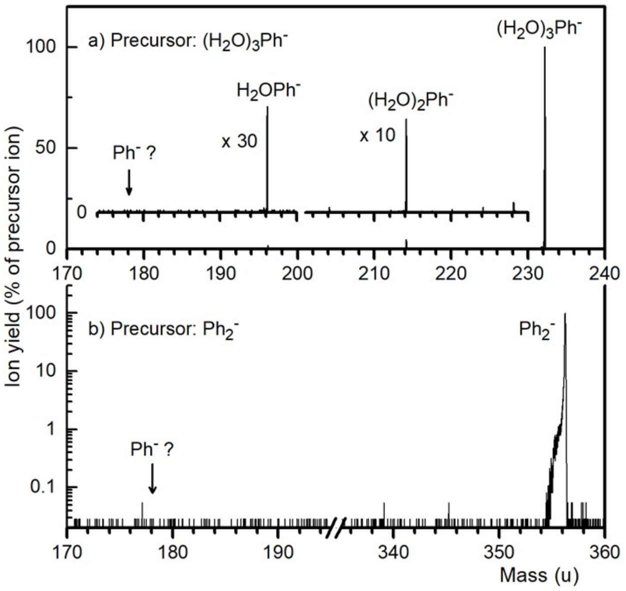

Fig. S2
Mass spectra of ions produced by collisions of $(H_2O)_3Ph^-$ and Ph_2^- with argon atoms at 5 eV ion energy (in the lab system) (panels a and b, respectively).

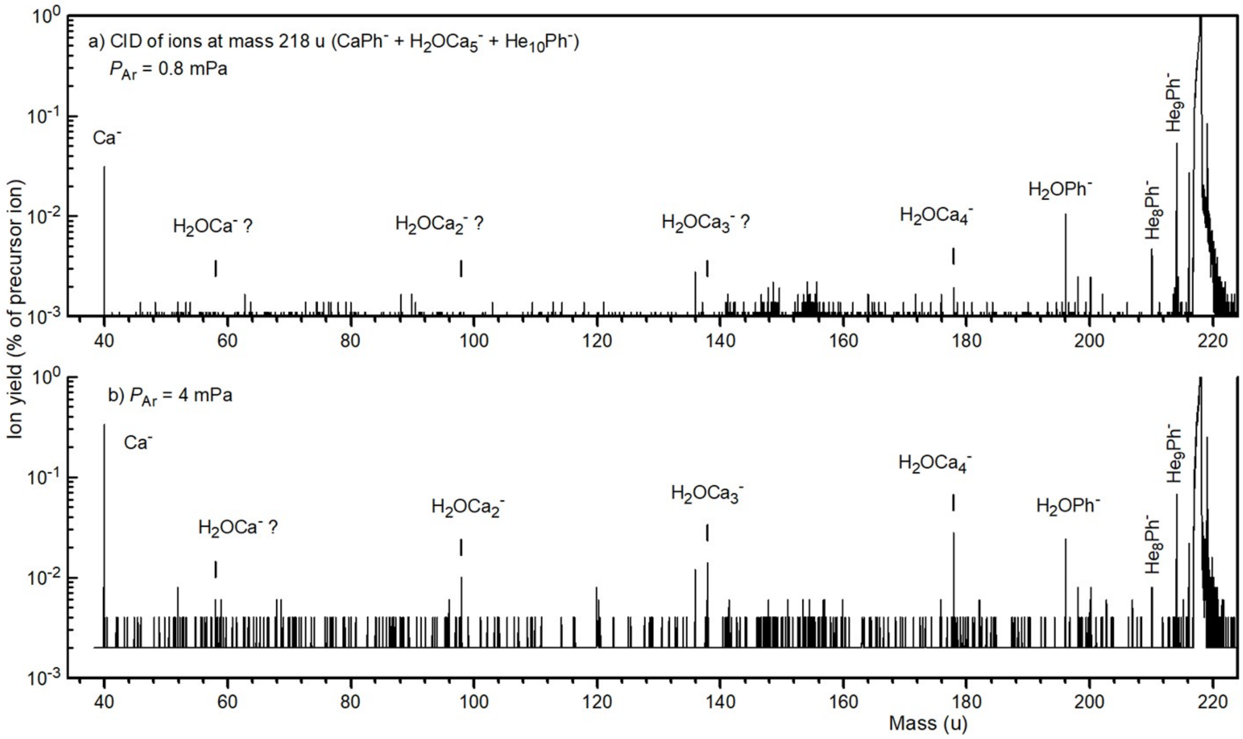

Fig. S3
Mass spectra of ions produced by collision of $CaPh^-$ with argon atoms at E = 5 eV ion energy (in the lab system) at two different argon pressures (panels a and b). The main product ion is Ca^-. A contamination of the parent ion mass peak at 218 u by $H_2OCa_5^-$ gives rise to a weak series of $H_2OCa_n^-$ product ions. He_8Ph^- and He_9Ph^- arise from a contamination by $He_{10}Ph^-$, and H_2OPh^- from a contamination by $He(H_2O)_2Ph^-$. Vertical bars indicate the expected position of ions; question marks flag ion peaks that are not positively identified.

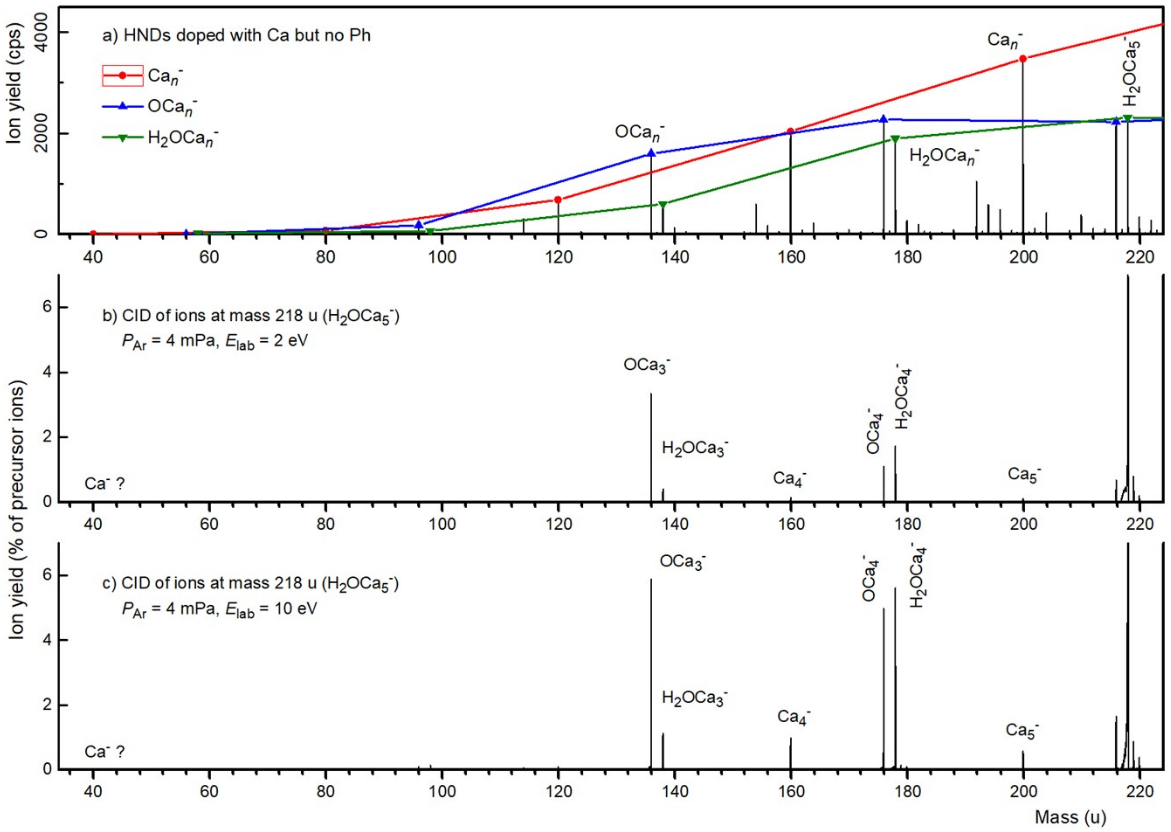

Fig. S4

Panel a: Negative ion mass spectrum of HNDs doped with Ca but no Ph. Panels b and c: CID spectra of mass 218 u anions (mostly $H_2OCa_5^-$ ions) with argon atoms at 2 and 10 eV, respectively. The main dissociation channels are loss of H_2O, H_2, and one or two Ca, but Ca^- is not detected.

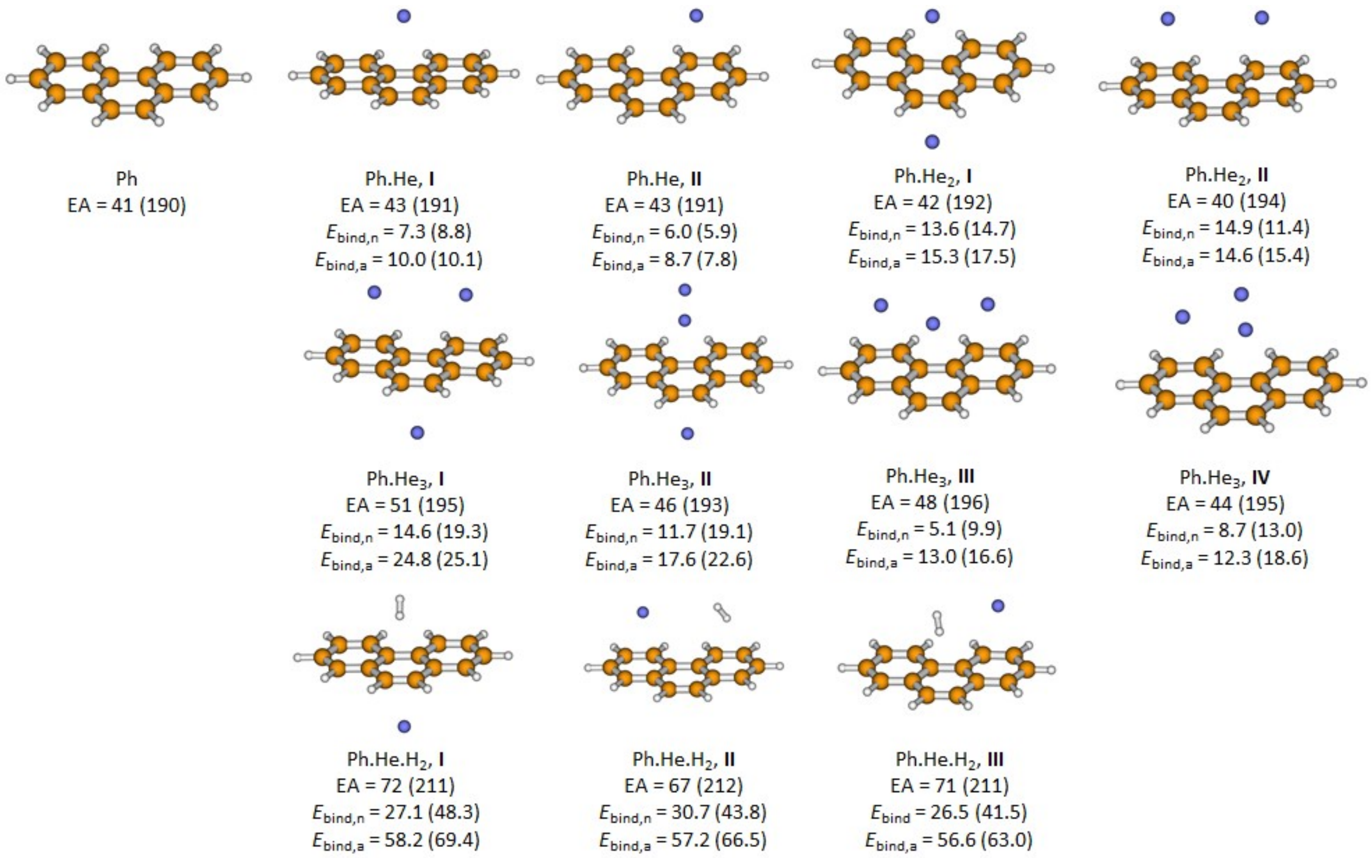

Fig. S5

Electron affinities, EA, and binding energies, E_{bind}, (both in meV) for phenanthrene complexed with He_n, n = 1-3, and $He.H_2$. Binding energies are given for both neutral ("n") and anionic ("a") form. Energies are given as calculated at the ωB97XD/aug-cc-pVDZ level; B3LYP-D3/aug-cc-pVDZ results are shown in parenthesis. Optimized structures of neutral molecules at the B3LYP-D3/aug-cc-pVDZ level are displayed.

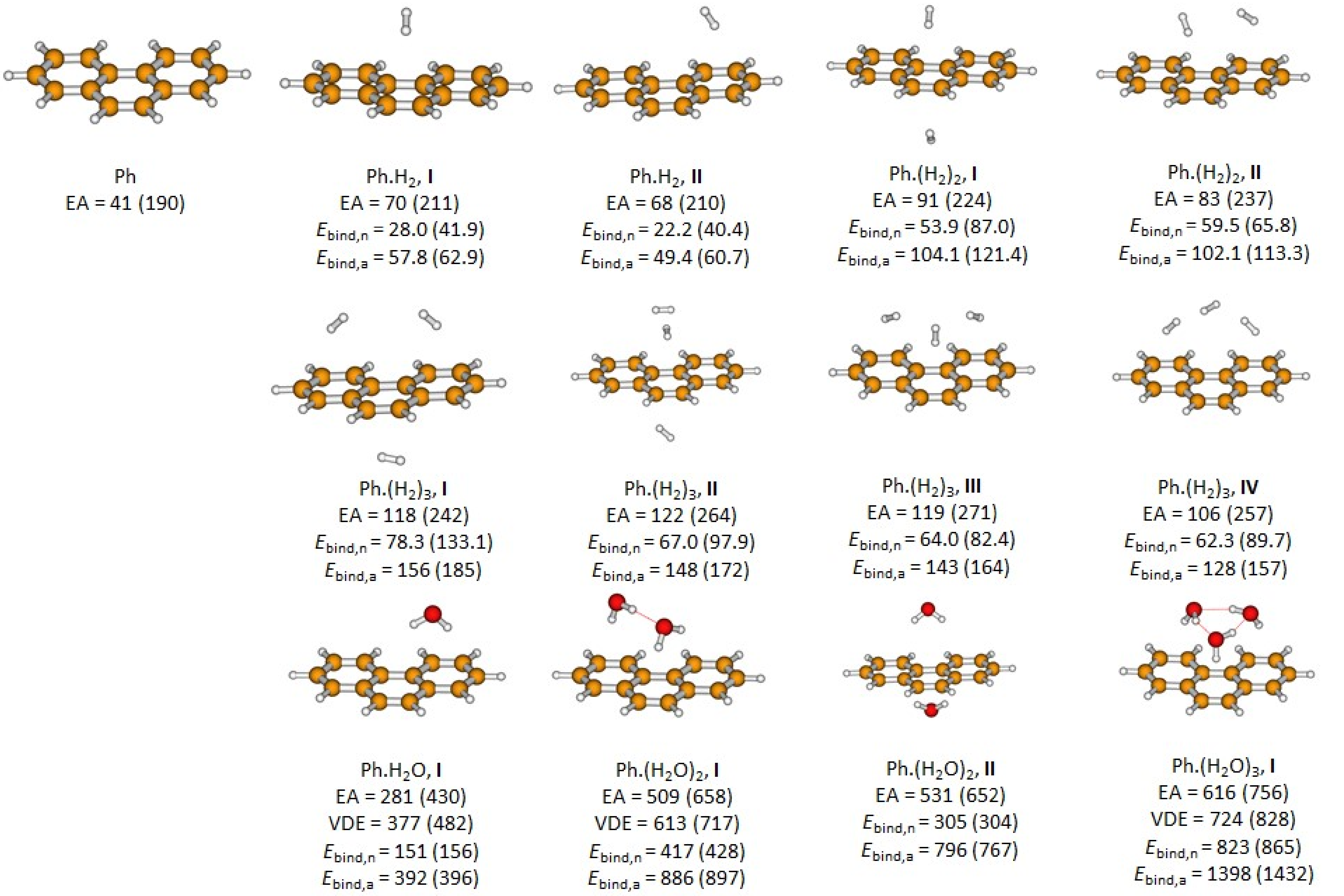

Fig. S6
Electron affinities, EA, and binding energies, E_{bind}, (both in meV) for phenanthrene complexed with $(H_2)_n$ and $(H_2O)_n$, n = 1-3. Binding energies are given for both neutral ("n") and anionic ("a") form. Energies are given as calculated at the ωB97XD/aug-cc-pVDZ level, B3LYP-D3/aug-cc-pVDZ results are shown in parenthesis. Optimized structures of neutral molecules at the B3LYP-D3/aug-cc-pVDZ level are displayed. For the most stable isomers with water molecules, the vertical detachment energy, VDE, is also given (in meV). Experimental vertical detachment energies of $(H_2O)_n$Ph are 270 ± 20, 510 ± 20, and 740 ± 20 meV for n = 1, 2, and 3, respectively.[1]

[1] Tschurl, M.; Boesl, U.; Gilb, S. The Electron Affinity of Phenanthrene. J. Chem. Phys. 2006, 125, 194310.

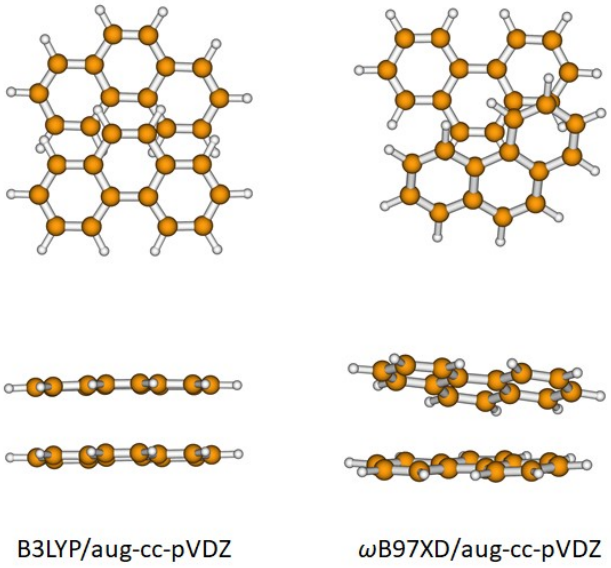

Fig. S7
Structure of Ph_2^- shown in top and side view as optimized at the B3LYP/aug-cc-pVDZ and ωB97XD/aug-cc-pVDZ levels.

Table S1

Benchmark of electron affinities (in meV) calculated using various DFT functionals. Both results for aug-cc-pVDZ ("aug-cc-pVDZ") and for optimization using aug-cc-pVDZ and single-point recalculation using aug-cc-pVTZ ("aug-cc-pVTZ//aug-cc-pVDZ") are given. In the latter case, the zero-point energy correction calculated using the aug-cc-pVDZ basis set was employed.

	aug-cc-pVDZ				aug-cc-pVTZ//aug-cc-pVDZ			
	Ph	Ph.He	Ph.H_2	Ph.H_2O	Ph	Ph.He	Ph.H_2	Ph.H_2O
B3LYP-D3	190	191	211	430	161	162	180	400
wB97XD	41	43	70	281	-15	-11	18	230
M062X-D3	99	103	119	339	101	106	125	348
M06-D3	250	255	254	494	120	126	141	370
M06L-D3	167	170	183	395	64	68	94	307
CAMB3LYP-D3	81	84	102	328	49	53	72	301

Table S2

Benchmark of vertical detachment energies (in meV) calculated using various DFT functionals. Both results for aug-cc-pVDZ ("aug-cc-pVDZ") and for optimization using aug-cc-pVDZ and single-point recalculation using aug-cc-pVTZ ("aug-cc-pVTZ//aug-cc-pVDZ") are given.

	aug-cc-pVDZ				aug-cc-pVTZ//aug-cc-pVDZ			
	Ph	Ph.He	Ph.H_2	Ph.H_2O	Ph	Ph.He	Ph.H_2	Ph.H_2O
B3LYP-D3	180	182	216	482	200	202	236	503
wB97XD	78	80	110	377	75	77	111	375
M062X-D3	136	140	168	419	180	185	216	471
M06-D3	246	252	279	531	175	180	216	458
M06L-D3	140	146	166	405	87	93	120	357
CAMB3LYP-D3	118	120	155	430	139	142	177	452

Table S3

Electron affinity (EA_{noZPE}) and vertical detachment energy (VDE_{noZPE}) without accounting for zero-point energy, electron affinity with included zero-point energy (EA_{ZPE}), and zero point energies for neutral and anionic complex (ZPE), all given for Ph and HePh in meV. Calculated at the ωB97XD/aug-cc-pVDZ level, B3LYP/aug-cc-pVDZ values are shown in parenthesis.

system (M)	EA_{noZPE}	VDE_{noZPE}	EA_{ZPE}	ZPE(M)	ZPE(M^-)
Ph	-113 (30)	78 (180)	41 (190)	5333 (5283)	5180 (5123)
HePh	-111 (32)	80 (182)	43 (191)	5341 (5289)	5186 (5131)

Cartesian coordinates of optimized molecules and ions (in Ångstrom) along with electronic energies including zero-point correction (in Hartree)

B3LYP minima

Ph2
E = -1078.853707
C -3.062367 2.187821 0.143857
C -1.648507 2.282815 0.125258
C -0.900922 1.502140 -0.807081
C -1.615820 0.646713 -1.680847
C -2.997123 0.570846 -1.646568
C -3.731304 1.349265 -0.728540
C 0.552116 1.610066 -0.818724
C 1.190817 2.495947 0.100296
C 0.397331 3.257272 1.024602
C -0.961390 3.154605 1.037213
C 2.603137 2.611708 0.095190
C 3.374583 1.877274 -0.786341
C 2.749425 0.994501 -1.690380
C 1.371627 0.865521 -1.702158
H -3.618680 2.792403 0.861627
H -4.819008 1.282354 -0.702350
H -3.517171 -0.107960 -2.321410
H -1.078734 0.020168 -2.388320
H -1.553515 3.739494 1.742372
H 0.907673 3.925423 1.719641
H 3.075355 3.294448 0.803031
H 4.460524 1.970938 -0.777040
H 3.353516 0.395703 -2.370804
H 0.921627 0.163253 -2.399188
C -0.587603 -0.447795 2.080913
C 0.769436 -0.344579 2.074129
C 1.569233 -1.121106 1.167437
C 0.935915 -2.002818 0.240281
C -0.518807 -2.113830 0.247786
C -1.269338 -1.337276 1.181631
C -1.231231 -2.964688 -0.632200
C -2.613618 -3.054018 -0.589341
C -3.347654 -2.289727 0.339736
C -2.680411 -1.444064 1.206706
C 1.760084 -2.735225 -0.648610
C 3.140262 -2.612484 -0.621076
C 3.759483 -1.746058 0.302059
C 2.980366 -1.012334 1.177829
H 1.312799 -3.415021 -1.370678
H 3.748876 -3.191382 -1.316167
H 4.845155 -1.649388 0.317836
H 3.445932 -0.325078 1.884327
H 1.274432 0.345100 2.750171
H -1.184512 0.158020 2.762318
H -3.237929 -0.833005 1.916535
H -4.435199 -2.358275 0.366174
H -3.134223 -3.720291 -1.277620
H -0.693247 -3.569858 -1.358802

Ph2-
E = -1078.871127
C 3.154094 2.857557 0.105475
C 3.166529 1.429181 0.097929
C 2.089688 0.732908 -0.554551
C 1.069229 1.497758 -1.158351
C 1.086458 2.892569 -1.154811
C 2.140619 3.571909 -0.509367
C 2.089687 -0.732909 -0.554552
C 3.166528 -1.429184 0.097929
C 4.209133 -0.692886 0.725745
C 4.209134 0.692883 0.725745
C 3.154091 -2.857559 0.105475
C 2.140615 -3.571910 -0.509366
C 1.086454 -2.892569 -1.154810
C 1.069228 -1.497758 -1.158351
H 3.969988 3.381951 0.607618
H 2.154348 4.663883 -0.489772
H 0.269110 3.446638 -1.613606
H 0.227341 0.996630 -1.629343
H 5.015549 1.243201 1.214861
H 5.015548 -1.243205 1.214861
H 3.969984 -3.381954 0.607618
H 2.154343 -4.663884 -0.489771
H 0.269105 -3.446637 -1.613606
H 0.227340 -0.996629 -1.629343
C -0.600180 0.691735 1.418409
C -0.600181 -0.691735 1.418409
C -1.654945 -1.426849 0.808900
C -2.747050 -0.734300 0.179188
C -2.747049 0.734302 0.179188
C -1.654944 1.426850 0.808900
C -3.765908 1.502712 -0.422077
C -3.736214 2.898908 -0.424165
C -2.657972 3.573569 0.181766
C -1.638419 2.852530 0.781300
C -3.765909 -1.502710 -0.422077
C -3.736216 -2.898905 -0.424166
C -2.657974 -3.573567 0.181766
C -1.638421 -2.852530 0.781299
H -4.605581 -1.003737 -0.904181
H -4.541959 -3.461675 -0.897781
H -2.621170 -4.664945 0.171099
H -0.790750 -3.367951 1.232982
H 0.232812 -1.240868 1.855787
H 0.232812 1.240867 1.855788
H -0.790748 3.367951 1.232983
H -2.621166 4.664947 0.171100
H -4.541956 3.461678 -0.897781
H -4.605580 1.003740 -0.904181

H2
E = -1.164101

H 0.000000 0.000000 0.380413
H 0.000000 0.000000 -0.380413

H2O
E = -76.423428
O -0.000000 0.000000 0.117806
H 0.000000 0.764220 -0.471226
H -0.000000 -0.764220 -0.471226

H2Ph, I
E = -540.583936
C -0.729250 -0.397352 -0.054504
C 0.729250 -0.397352 -0.054504
H -1.005825 -2.554782 0.000453
C 0.681350 2.079414 -0.113951
C -0.681350 2.079414 -0.113951
H 1.005825 -2.554782 0.000453
C -1.500115 -1.585845 -0.022910
C 1.424502 0.849624 -0.084568
C -1.424502 0.849623 -0.084568
C 1.500115 -1.585845 -0.022910
C -2.885082 -1.549293 -0.020616
C 2.841355 0.863191 -0.080942
C -2.841355 0.863191 -0.080942
C 2.885082 -1.549293 -0.020616
C -3.566000 -0.314559 -0.049513
C 3.566000 -0.314559 -0.049513
H 1.233891 3.019569 -0.135217
H -1.233891 3.019568 -0.135217
H -3.450963 -2.480917 0.004249
H 3.356160 1.824838 -0.102920
H -3.356160 1.824838 -0.102920
H 3.450963 -2.480917 0.004248
H -4.656005 -0.290871 -0.046850
H 4.656005 -0.290871 -0.046851
H -0.000003 1.077843 2.571717
H 0.000004 0.544333 3.112907

H2Ph-, I
E = -540.591674
C -0.740117 -0.402775 -0.046100
C 0.740117 -0.402775 -0.046100
H -0.996544 -2.544353 0.032580
C 0.705644 2.075514 -0.135758
C -0.705644 2.075514 -0.135759
H 0.996544 -2.544353 0.032580
C -1.502861 -1.579598 -0.003079
C 1.435275 0.869683 -0.093084
C -1.435275 0.869683 -0.093084
C 1.502860 -1.579598 -0.003080
C -2.910189 -1.575305 -0.003692
C 2.871974 0.847881 -0.092330
C -2.871974 0.847881 -0.092330
C 2.910188 -1.575305 -0.003692
C -3.585296 -0.339215 -0.048782
C 3.585296 -0.339215 -0.048782
H 1.253586 3.019910 -0.168040
H -1.253586 3.019910 -0.168040
H -3.465477 -2.513744 0.031120
H 3.402334 1.802945 -0.126223
H -3.402334 1.802946 -0.126223
H 3.465477 -2.513744 0.031119
H -4.678744 -0.314580 -0.049363
H 4.678744 -0.314580 -0.049363
H 0.000004 1.318807 2.464440
H 0.000002 1.026619 3.169318

H2Ph, II
E = -540.583884
C -3.524757 -0.282991 -0.135987
C -2.797120 0.893530 -0.115847
C -1.380587 0.876471 -0.090997
C -0.689111 -0.372906 -0.086865
C -1.462503 -1.559730 -0.110482
C -2.847213 -1.519848 -0.133961
C -0.634594 2.104673 -0.062728
C 0.727215 2.100853 -0.030187
C 1.467322 0.868510 -0.023091
C 0.769141 -0.377125 -0.051685
C 2.883840 0.877755 0.012211
C 3.605214 -0.302577 0.019309
C 2.921399 -1.535634 -0.008122
C 1.536664 -1.568196 -0.042900
H 1.040126 -2.536039 -0.063514
H -0.970614 -2.530024 -0.106224
H -1.184769 3.046473 -0.066709
H 1.282130 3.039668 -0.008120
H 3.484656 -2.469136 -0.002187
H -3.309553 1.856577 -0.115609
H 3.401057 1.838135 0.033534
H -3.415354 -2.450198 -0.148681
H 4.694963 -0.281959 0.046569
H -4.614488 -0.256421 -0.152368
H -2.110903 -0.244367 2.542169
H -1.746709 -0.229415 3.209129

H2Ph-, II
E = -540.591594
C -3.545978 -0.290913 -0.137003
C -2.828121 0.893747 -0.104883
C -1.390954 0.910403 -0.081032
C -0.701811 -0.366676 -0.083867
C -1.469495 -1.541108 -0.117378
C -2.876799 -1.530878 -0.145922
C -0.656426 2.112906 -0.050897
C 0.753692 2.107001 -0.022893
C 1.477964 0.897523 -0.020821
C 0.777659 -0.372995 -0.050489
C 2.914398 0.868894 0.010209
C 3.622313 -0.322010 0.012159

C 2.941959 -1.556153 -0.015236
C 1.535174 -1.554047 -0.045640
H 1.024854 -2.517168 -0.066128
H -0.967084 -2.508412 -0.118889
H -1.200571 3.060214 -0.049137
H 1.305451 3.049628 0.000276
H 3.493094 -2.497636 -0.012586
H -3.354837 1.851232 -0.095413
H 3.448827 1.822074 0.032332
H -3.435792 -2.467275 -0.167705
H 4.715613 -0.302417 0.036145
H -4.639067 -0.261188 -0.152769
H -1.791357 -0.383801 2.482120
H -1.920583 -0.379420 3.233914

H2OPh, I
E = -615.847472
C -2.630619 0.946812 -0.529848
C -1.219570 0.915190 -0.401092
C -0.538967 -0.341112 -0.392350
C -1.316613 -1.519254 -0.520532
C -2.695524 -1.465278 -0.643517
C -3.363022 -0.221754 -0.646399
C 0.911631 -0.361888 -0.235730
C 1.612699 0.875120 -0.098559
C 0.883943 2.114146 -0.118204
C -0.470413 2.133863 -0.262940
C 3.021080 0.868739 0.057742
C 3.730534 -0.318430 0.080183
C 3.043475 -1.542802 -0.053331
C 1.666842 -1.560712 -0.207439
H -3.135321 1.913576 -0.527689
H -4.448375 -0.184156 -0.739863
H -3.267460 -2.388703 -0.733742
H -0.833542 -2.493647 -0.514030
H -1.012169 3.080157 -0.270868
H 1.441279 3.045473 -0.010995
H 3.541133 1.822090 0.161141
H 4.813916 -0.310070 0.201517
H 3.597931 -2.481249 -0.034923
H 1.167523 -2.522064 -0.307134
H -0.915880 -0.130998 2.416024
O -1.800657 -0.268537 2.775531
H -2.356648 -0.337951 1.988415

H2OPh-, I
E = -615.863260
C -2.694413 0.224738 -0.692352
C -1.276512 0.434405 -0.585661
C -0.457470 -0.682485 -0.159638
C -1.087640 -1.901791 0.129586
C -2.481077 -2.083456 0.020443
C -3.276054 -1.001683 -0.395066
C 1.004117 -0.482252 -0.042640
C 1.555437 0.828575 -0.329106
C 0.708404 1.884272 -0.722790
C -0.684622 1.690085 -0.850641
C 2.974950 1.000674 -0.200386
C 3.805620 -0.039406 0.178676
C 3.269920 -1.315587 0.452650
C 1.883899 -1.508134 0.336443
H -3.317270 1.061150 -1.017495
H -4.358703 -1.123233 -0.487804
H -2.931595 -3.047735 0.258927
H -0.487307 -2.751002 0.455391
H -1.323504 2.509732 -1.186844
H 1.147507 2.861085 -0.936065
H 3.395906 1.986741 -0.411930
H 4.882269 0.130343 0.265687
H 3.918759 -2.140163 0.750893
H 1.487417 -2.500068 0.553308
H -1.490615 2.223880 1.345697
O -2.216216 2.144476 1.987888
H -2.660480 1.345740 1.670032

H2HePh, II
E = -543.493103
C 3.565265 -0.283095 -0.124649
C 2.840245 0.894739 -0.101975
C 1.423469 0.881094 -0.106968
C 0.728816 -0.366314 -0.135682
C 1.499955 -1.554700 -0.158454
C 2.884923 -1.517945 -0.153013
C 0.679548 2.110816 -0.081045
C -0.682661 2.110359 -0.081570
C -1.425195 0.880028 -0.108007
C -0.729801 -0.366804 -0.136578
C -2.841910 0.892364 -0.099027
C -3.566032 -0.286373 -0.117188
C -2.884606 -1.520750 -0.147487
C -1.499609 -1.555945 -0.157292
He 1.939289 -0.328417 2.878398
H -1.004639 -2.524451 -0.177303
H 1.006057 -2.523806 -0.180197
H 1.231833 3.051201 -0.060035
H -1.235756 3.050218 -0.060645
H -3.449893 -2.452845 -0.161027
H 3.354734 1.856519 -0.079725
H -3.357329 1.853494 -0.074164
H 3.451059 -2.449539 -0.170651
H -4.655921 -0.263411 -0.107461
H 4.655239 -0.259135 -0.120087
H -2.126290 -0.311863 2.529420
H -1.702111 -0.274387 3.158686

H2HePh-, II
E = -543.500901
C 3.579913 -0.297162 -0.137733
C 2.867230 0.890306 -0.106335
C 1.430473 0.912824 -0.103069

C 0.734976 -0.360061 -0.135748
C 1.497300 -1.537508 -0.166560
C 2.904268 -1.533762 -0.167498
C 0.701319 2.118885 -0.068876
C -0.709116 2.118893 -0.063980
C -1.439077 0.913585 -0.094424
C -0.744797 -0.360067 -0.132371
C -2.876304 0.890772 -0.084398
C -3.589695 -0.296522 -0.116190
C -2.915670 -1.533218 -0.158393
C -1.508097 -1.537495 -0.163741
He 2.062032 -0.393833 2.870249
H -1.001585 -2.502356 -0.190493
H 0.990564 -2.502492 -0.189854
H 1.249479 3.063572 -0.044785
H -1.256966 3.063610 -0.035347
H -3.471106 -2.471722 -0.179722
H 3.397912 1.845486 -0.082488
H -3.406675 1.845626 -0.048801
H 3.459246 -2.472649 -0.191464
H -4.682958 -0.271815 -0.105490
H 4.673334 -0.273150 -0.138167
H -1.776923 -0.434074 2.454274
H -1.894717 -0.439184 3.207724

H2HePh, III
E = -543.493020
C 3.610304 -0.332770 -0.020928
C 2.895181 0.850659 -0.057925
C 1.478927 0.847792 -0.097608
C 0.774201 -0.394166 -0.098988
C 1.535287 -1.588709 -0.061114
C 2.919989 -1.562584 -0.022887
C 0.745768 2.083391 -0.129888
C -0.616515 2.093775 -0.160976
C -1.369168 0.869296 -0.166993
C -0.683860 -0.383208 -0.136863
C -2.785440 0.893296 -0.204635
C -3.518779 -0.279334 -0.216251
C -2.847751 -1.519289 -0.187708
C -1.463764 -1.566208 -0.147667
H -0.977152 -2.539023 -0.125859
H 1.033550 -2.554087 -0.061018
H 1.305510 3.019478 -0.126162
H -1.161532 3.038270 -0.183588
H -3.420577 -2.446918 -0.196100
H 3.417310 1.808594 -0.055743
H -3.292553 1.858997 -0.226313
H 3.478283 -2.498640 0.006353
H -4.608123 -0.247533 -0.246650
H 4.700017 -0.317249 0.010006
H 0.096933 1.112558 2.536499
H 0.215124 0.596445 3.081313
He -2.416534 -0.451266 2.924926

H2HePh-, III
E = -543.500773
C 3.622415 -0.359502 -0.032556
C 2.918186 0.832797 -0.083708
C 1.482178 0.864410 -0.115876
C 0.777671 -0.403351 -0.091531
C 1.531264 -1.585762 -0.039678
C 2.938270 -1.591124 -0.010556
C 0.761462 2.075734 -0.165550
C -0.649214 2.085703 -0.197764
C -1.387513 0.884814 -0.177562
C -0.702156 -0.392733 -0.124294
C -2.823951 0.872620 -0.209682
C -3.545881 -0.309796 -0.192988
C -2.880226 -1.550684 -0.142031
C -1.473519 -1.564517 -0.108597
H -0.974559 -2.532898 -0.068753
H 1.017773 -2.547191 -0.021300
H 1.316609 3.016302 -0.181293
H -1.189790 3.034117 -0.235705
H -3.442324 -2.485557 -0.127797
H 3.455602 1.784351 -0.099355
H -3.346944 1.831508 -0.248742
H 3.486329 -2.533541 0.030238
H -4.638788 -0.277705 -0.218668
H 4.715738 -0.342316 -0.009185
H 0.350944 1.406656 2.441472
H 0.268653 1.165288 3.160927
He -2.216578 -0.335332 2.866201

H2HePh, I
E = -543.493269
C -3.565898 -0.327362 -0.002504
C -2.841261 0.850765 0.001279
C -1.424390 0.837219 0.002379
C -0.729179 -0.410071 -0.001646
C -1.500028 -1.598963 -0.005196
C -2.884948 -1.562462 -0.005430
C -0.681314 2.067262 0.005222
C 0.681314 2.067262 0.005221
C 1.424391 0.837219 0.002378
C 0.729179 -0.410071 -0.001647
C 2.841261 0.850764 0.001277
C 3.565898 -0.327364 -0.002506
C 2.884948 -1.562463 -0.005432
C 1.500027 -1.598964 -0.005197
H 1.005757 -2.568167 -0.008161
H -1.005758 -2.568167 -0.008160
H -1.233849 3.007585 0.006875
H 1.233850 3.007585 0.006875
H 3.450841 -2.494350 -0.008427
H -3.356060 1.812646 0.003029
H 3.356061 1.812644 0.003027
H -3.450842 -2.494348 -0.008423
H 4.655908 -0.303771 -0.003340

H -4.655908 -0.303769 -0.003336
H 0.000145 1.003903 -2.658241
H -0.000162 0.526362 -3.249381
He 0.000010 0.642615 2.999241

H2HePh-, I
E = -543.501010
C -3.585151 -0.351990 -0.003522
C -2.871878 0.835802 0.011362
C -1.435189 0.857568 0.009590
C -0.740049 -0.415499 -0.009123
C -1.502722 -1.593037 -0.023527
C -2.910001 -1.588791 -0.021080
C -0.705586 2.063946 0.024884
C 0.705585 2.063945 0.024884
C 1.435188 0.857567 0.009590
C 0.740048 -0.415500 -0.009123
C 2.871877 0.835802 0.011363
C 3.585151 -0.351991 -0.003521
C 2.910000 -1.588791 -0.021079
C 1.502722 -1.593038 -0.023526
H 0.996393 -2.558313 -0.037615
H -0.996394 -2.558313 -0.037616
H -1.253532 3.008748 0.036718
H 1.253532 3.008747 0.036718
H 3.465245 -2.527758 -0.033155
H -3.402263 1.791347 0.024102
H 3.402263 1.791346 0.024102
H -3.465246 -2.527758 -0.033157
H 4.678591 -0.327416 -0.001788
H -4.678591 -0.327415 -0.001789
H 0.000019 1.251717 -2.565077
H 0.000012 0.962188 -3.270843
He 0.000000 0.658464 2.998185

HePh, I
E = -542.327715
C 0.394917 -0.055331 0.729421
C 0.394917 -0.055331 -0.729421
H 2.552511 -0.005367 1.006282
C -2.082342 -0.111362 -0.681125
C -2.082342 -0.111362 0.681125
H 2.552511 -0.005367 -1.006282
C 1.583498 -0.027288 1.500366
C -0.852475 -0.083525 -1.424488
C -0.852475 -0.083525 1.424488
C 1.583498 -0.027288 -1.500366
C 1.547019 -0.027238 2.885435
C -0.865689 -0.082885 -2.841452
C -0.865689 -0.082885 2.841452
C 1.547019 -0.027238 -2.885435
C 0.312258 -0.055223 3.566199
C 0.312258 -0.055223 -3.566199
H -3.022460 -0.132316 -1.233852
H -3.022460 -0.132316 1.233852
H 2.478726 -0.005345 3.451313
H -1.827332 -0.104681 -3.356289
H -1.827332 -0.104681 3.356289
H 2.478726 -0.005345 -3.451313
H 0.288337 -0.054991 4.656225
H 0.288337 -0.054991 -4.656225
He -0.692893 2.959817 -0.000000

HePh-, I
E = -542.334727
C 0.394267 -0.053985 0.740050
C 0.394267 -0.053985 -0.740050
H 2.536633 -0.000306 0.996773
C -2.084896 -0.114681 -0.705277
C -2.084896 -0.114681 0.705277
H 2.536633 -0.000306 -0.996773
C 1.571386 -0.024133 1.503016
C -0.879221 -0.085292 -1.435081
C -0.879221 -0.085292 1.435081
C 1.571386 -0.024133 -1.503016
C 1.567158 -0.023682 2.910707
C -0.857193 -0.084378 -2.872265
C -0.857193 -0.084378 2.872265
C 1.567158 -0.023682 -2.910707
C 0.330366 -0.054359 3.585607
C 0.330366 -0.054359 -3.585607
H -3.029798 -0.137561 -1.253260
H -3.029798 -0.137561 1.253260
H 2.505914 0.000099 3.466078
H -1.812589 -0.108034 -3.402753
H -1.812589 -0.108034 3.402753
H 2.505914 0.000099 -3.466078
H 0.305652 -0.054549 4.679120
H 0.305652 -0.054549 -4.679120
He -0.757020 2.943410 -0.000000

HePh, II
E = -542.327611
C -1.463162 -1.559317 -0.111076
C -0.689285 -0.372656 -0.084112
C -1.380752 0.876927 -0.088949
C -2.797226 0.894292 -0.119543
C -3.524923 -0.281808 -0.144858
C -2.847732 -1.518779 -0.140694
C -0.634174 2.104990 -0.061011
C 0.727756 2.101071 -0.029274
C 1.467686 0.868628 -0.022574
C 0.769257 -0.376998 -0.050006
C 1.536783 -1.568198 -0.042245
C 2.921563 -1.535791 -0.009321
C 3.605550 -0.302773 0.017449
C 2.884307 0.877621 0.010824
H 3.401688 1.837947 0.031346
H 4.695341 -0.282298 0.043263
H 3.484739 -2.469363 -0.004158

H 1.040013 -2.535967 -0.062449
H 1.282814 3.039834 -0.008100
H -1.184115 3.046994 -0.065707
H -3.309315 1.857618 -0.122311
H -4.614609 -0.254987 -0.167908
H -3.416061 -2.449019 -0.160504
H -0.971557 -2.529887 -0.108363
He -1.931413 -0.252061 2.938621

HePh-, II
E = -542.334644
C -1.464467 -1.543033 -0.115345
C -0.697626 -0.368585 -0.085067
C -1.387866 0.907827 -0.087306
C -2.824616 0.891232 -0.121304
C -3.541965 -0.293882 -0.149654
C -2.871730 -1.533475 -0.146844
C -0.653915 2.111043 -0.056267
C 0.756299 2.105688 -0.023218
C 1.481596 0.896835 -0.018718
C 0.782118 -0.374370 -0.048943
C 1.540601 -1.554770 -0.042820
C 2.947894 -1.555980 -0.008997
C 3.627079 -0.321496 0.020046
C 2.918278 0.869164 0.015468
H 3.452088 1.822726 0.038187
H 4.720361 -0.301035 0.046509
H 3.499691 -2.497128 -0.005389
H 1.030994 -2.518279 -0.065116
H 1.307495 3.048734 0.000159
H -1.198343 3.058303 -0.059131
H -3.351491 1.848861 -0.123209
H -4.635065 -0.264975 -0.174199
H -3.430299 -2.470346 -0.169035
H -0.961791 -2.510397 -0.113887
He -2.051858 -0.316828 2.919464

(H2)2Ph, II
E = -541.748918
C 3.595190 -0.338293 -0.051198
C 2.879684 0.844511 -0.099233
C 1.463280 0.841030 -0.125594
C 0.758638 -0.400693 -0.102848
C 1.520047 -1.594531 -0.054223
C 2.905037 -1.567970 -0.029016
C 0.729721 2.076262 -0.164043
C -0.632710 2.086484 -0.177352
C -1.385320 0.862026 -0.161809
C -0.699750 -0.390015 -0.126291
C -2.801619 0.885808 -0.182617
C -3.535045 -0.286942 -0.171976
C -2.863485 -1.526734 -0.138801
C -1.479034 -1.573031 -0.115861
H -0.992018 -2.545353 -0.087813
H 1.018522 -2.559823 -0.035571
H 1.289470 3.012226 -0.177713
H -1.178011 3.030725 -0.203731
H -3.436206 -2.454331 -0.129669
H 3.401626 1.802380 -0.114781
H -3.309120 1.851131 -0.206730
H 3.463618 -2.503501 0.009375
H -4.624632 -0.255442 -0.188188
H 4.685126 -0.322362 -0.030281
H 0.455034 1.179433 2.600154
H 0.716370 0.799834 3.203870
H -1.851579 -0.142443 3.030915
H -2.366003 -0.399950 2.535341

(H2)2Ph-, II
E = -541.757626
C 3.607649 -0.376514 -0.055727
C 2.907884 0.817724 -0.112414
C 1.472338 0.854771 -0.134651
C 0.762985 -0.409492 -0.093658
C 1.512163 -1.594574 -0.034887
C 2.918434 -1.605347 -0.016831
C 0.756356 2.068888 -0.188727
C -0.654004 2.083916 -0.208090
C -1.396827 0.886340 -0.171648
C -0.716573 -0.393723 -0.116809
C -2.833709 0.879109 -0.185451
C -3.560074 -0.300678 -0.161332
C -2.899470 -1.544132 -0.117962
C -1.492342 -1.563125 -0.093430
H -0.996643 -2.532941 -0.052497
H 0.994950 -2.553597 -0.002465
H 1.315147 3.006851 -0.218766
H -1.191419 3.034013 -0.247151
H -3.464838 -2.476598 -0.094623
H 3.448816 1.766947 -0.139818
H -3.353569 1.839782 -0.217174
H 3.462988 -2.549456 0.029806
H -4.652903 -0.263886 -0.172861
H 4.701092 -0.363630 -0.040385
H 0.668569 1.609682 2.454937
H 0.625148 1.433586 3.196922
H -2.000669 -0.348412 3.200256
H -1.865547 -0.421315 2.453519

(H2)2Ph, I
E = -541.749696
C 3.565699 -0.329381 0.000007
C 2.841050 0.848653 0.000003
C 1.424318 0.834820 -0.000001
C 0.729027 -0.412184 -0.000001
C 1.499796 -1.601019 0.000003
C 2.884649 -1.564442 0.000006
C 0.681541 2.064818 -0.000003
C -0.681539 2.064818 -0.000005
C -1.424316 0.834821 -0.000005

C -0.729026 -0.412184 -0.000004
C -2.841047 0.848656 -0.000004
C -3.565698 -0.329377 -0.000003
C -2.884649 -1.564439 -0.000004
C -1.499796 -1.601017 -0.000004
H -1.005630 -2.570229 -0.000004
H 1.005629 -2.570230 0.000004
H 1.233999 3.005100 -0.000001
H -1.233996 3.005100 -0.000003
H -3.450624 -2.496285 -0.000004
H 3.355736 1.810568 0.000003
H -3.355732 1.810571 -0.000004
H 3.450622 -2.496289 0.000010
H -4.655670 -0.305799 -0.000003
H 4.655671 -0.305804 0.000010
H 0.000345 1.029278 2.674387
H -0.000462 0.479767 3.198989
H 0.000032 1.029305 -2.674405
H 0.000031 0.479685 -3.198893

(H2)2Ph-, I
E = -541.757926
C -3.584781 -0.359360 0.000033
C -2.871596 0.828520 0.000009
C -1.435391 0.850110 0.000002
C -0.740118 -0.422565 0.000010
C -1.502502 -1.600339 0.000036
C -2.909413 -1.596019 0.000051
C -0.705971 2.057136 -0.000012
C 0.705975 2.057138 0.000010
C 1.435397 0.850114 -0.000003
C 0.740127 -0.422562 -0.000011
C 2.871603 0.828527 -0.000007
C 3.584791 -0.359350 -0.000030
C 2.909425 -1.596012 -0.000050
C 1.502515 -1.600335 -0.000037
H 0.996093 -2.565546 -0.000051
H -0.996078 -2.565549 0.000048
H -1.253843 3.001743 -0.000010
H 1.253844 3.001746 0.000007
H 3.464598 -2.535083 -0.000071
H -3.401939 1.784081 -0.000003
H 3.401943 1.784089 0.000006
H -3.464584 -2.535091 0.000072
H 4.678157 -0.334872 -0.000035
H -4.678148 -0.334884 0.000041
H -0.000768 1.210258 -2.575690
H -0.000339 0.894412 -3.269902
H 0.000517 1.210254 2.575689
H 0.000180 0.894422 3.269907

(H2O)2Ph, II
E = -692.276330
C 1.499459 1.251419 -0.000001
C 0.729561 0.059436 0.000000
C 1.424539 -1.188351 0.000002
C 2.841120 -1.200111 0.000001
C 3.565531 -0.021289 -0.000001
C 2.885735 1.214066 -0.000002
C 0.681029 -2.418657 0.000005
C -0.681030 -2.418657 0.000006
C -1.424540 -1.188351 0.000003
C -0.729561 0.059436 0.000000
C -1.499459 1.251420 -0.000003
C -2.885735 1.214067 -0.000003
C -3.565531 -0.021288 0.000001
C -2.841121 -1.200110 0.000004
O 0.000000 1.568628 -3.053042
H -3.356847 -2.161184 0.000006
H -4.655226 -0.044948 0.000001
H -3.450568 2.146057 -0.000006
H -1.005312 2.220011 -0.000006
H -1.233539 -3.358779 0.000008
H 1.233537 -3.358779 0.000006
H 3.356846 -2.161185 0.000003
H 4.655225 -0.044949 -0.000003
H 3.450568 2.146056 -0.000003
H 1.005312 2.220011 -0.000002
H 0.760318 1.235325 -2.559866
H -0.760318 1.235321 -2.559868
H 0.760319 1.235336 2.559859
O 0.000003 1.568646 3.053034
H -0.760317 1.235341 2.559862

(H2O)2Ph-, II
E = -692.300300
C 1.502774 -1.247607 0.000005
C 0.739096 -0.065894 0.000001
C 1.433696 1.207549 -0.000003
C 2.871176 1.184249 -0.000002
C 3.583752 -0.002523 0.000002
C 2.910800 -1.241569 0.000006
C 0.704457 2.411965 -0.000006
C -0.704457 2.411965 -0.000005
C -1.433696 1.207549 -0.000002
C -0.739096 -0.065894 0.000000
C -1.502774 -1.247607 0.000001
C -2.910800 -1.241569 0.000002
C -3.583752 -0.002523 0.000001
C -2.871176 1.184249 -0.000002
O 0.000001 -1.510327 2.974967
H -3.401539 2.139272 -0.000003
H -4.676534 0.022419 0.000001
H -3.465424 -2.179784 0.000003
H -0.997461 -2.212837 0.000002
H -1.251852 3.356751 -0.000007
H 1.251852 3.356751 -0.000008
H 3.401539 2.139272 -0.000005
H 4.676534 0.022419 0.000003
H 3.465424 -2.179784 0.000010

H 0.997461 -2.212837 0.000008
H 0.753026 -1.260069 2.419735
H -0.753025 -1.260071 2.419734
H 0.753025 -1.260086 -2.419735
O -0.000001 -1.510345 -2.974966
H -0.753026 -1.260085 -2.419735

(H2O)2Ph, I
E = -692.280877
C -1.412925 -1.322728 0.965258
C -0.576287 -0.214185 0.685194
C -1.192573 1.061957 0.511712
C -2.600315 1.182592 0.613011
C -3.391554 0.081091 0.883595
C -2.788637 -1.181480 1.063572
C -0.378705 2.214005 0.230596
C 0.977878 2.112718 0.130179
C 1.641733 0.845604 0.284463
C 0.872290 -0.328005 0.554258
C 1.561174 -1.561424 0.664389
C 2.937620 -1.635042 0.521035
C 3.692035 -0.472016 0.261768
C 3.048498 0.747233 0.146128
H 3.619792 1.653258 -0.059928
H 4.774478 -0.535801 0.150646
H 3.439117 -2.598914 0.608361
H 1.009011 -2.477698 0.860385
H 1.584241 2.995668 -0.074807
H -0.870627 3.179259 0.105685
H -3.054768 2.163432 0.468958
H -4.474203 0.184128 0.952959
H -3.407408 -2.053333 1.275005
H -0.981627 -2.311804 1.100008
H -2.256275 -1.233910 -1.554264
O -2.074367 -1.314568 -2.498003
H -1.282405 -0.766854 -2.638744
H 0.251324 1.061979 -2.160567
O 0.357395 0.288215 -2.731053
H 1.043734 -0.230504 -2.292187

(H2O)2Ph-, I
E = -692.305067
C 1.306739 -1.422426 -0.863363
C 0.490996 -0.300307 -0.658326
C 1.109606 1.009524 -0.639268
C 2.529248 1.083891 -0.827094
C 3.301096 -0.051112 -1.013865
C 2.700349 -1.325582 -1.032735
C 0.320364 2.164659 -0.431206
C -1.084537 2.075122 -0.298989
C -1.736093 0.825403 -0.288116
C -0.969295 -0.398794 -0.433916
C -1.646545 -1.625859 -0.352674
C -3.036669 -1.714511 -0.165431
C -3.784030 -0.525291 -0.044252
C -3.155349 0.707158 -0.100414
H -3.739643 1.624192 0.004896
H -4.866221 -0.574392 0.102329
H -3.524286 -2.688138 -0.107789
H -1.083235 -2.554273 -0.442695
H -1.680087 2.980116 -0.165656
H 0.809184 3.140728 -0.434002
H 3.002310 2.068000 -0.804550
H 4.382241 0.043495 -1.140356
H 3.300450 -2.222901 -1.184984
H 0.859429 -2.415611 -0.879178
H 2.581339 -1.093564 1.511805
O 2.463008 -0.998238 2.467714
H 1.683932 -0.415269 2.541783
H 0.270748 1.341201 1.812792
O 0.099553 0.701374 2.526837
H -0.571928 0.130075 2.127107

He2Ph, II
E = -545.236907
C -2.841413 0.893094 -0.105087
C -1.424573 0.879981 -0.107791
C -0.729450 -0.367315 -0.133950
C -1.500236 -1.556050 -0.156676
C -2.885235 -1.519690 -0.153462
C -3.566020 -0.284985 -0.127276
C 0.729450 -0.367315 -0.133950
C 1.424573 0.879981 -0.107791
C 0.681150 2.110024 -0.082930
C -0.681150 2.110024 -0.082930
C 2.841413 0.893094 -0.105088
C 3.566019 -0.284985 -0.127276
C 2.885234 -1.519691 -0.153462
C 1.500236 -1.556051 -0.156676
He 1.922556 -0.298722 2.911908
H -3.356279 1.854723 -0.084587
H -4.656015 -0.261400 -0.124515
H -3.451076 -2.451483 -0.170947
H -1.005876 -2.524994 -0.176941
H -1.233793 3.050252 -0.063786
H 1.233794 3.050252 -0.063786
H 3.356280 1.854723 -0.084587
H 4.656015 -0.261400 -0.124515
H 3.451076 -2.451483 -0.170947
H 1.005876 -2.524994 -0.176941
He -1.922555 -0.298726 2.911904

He2Ph-, II
E = -545.244018
C -2.872167 0.888600 -0.102533
C -1.435104 0.910798 -0.102872
C -0.740074 -0.362600 -0.134570
C -1.502827 -1.539776 -0.161896
C -2.910416 -1.535728 -0.159239
C -3.585362 -0.299028 -0.129400

C 0.740074 -0.362600 -0.134570
C 1.435104 0.910798 -0.102872
C 0.705298 2.116559 -0.072894
C -0.705298 2.116559 -0.072894
C 2.872167 0.888600 -0.102534
C 3.585362 -0.299028 -0.129400
C 2.910416 -1.535728 -0.159238
C 1.502827 -1.539776 -0.161895
He 2.019917 -0.362210 2.901031
H -3.402666 1.843930 -0.078934
H -4.678831 -0.274421 -0.127122
H -3.465690 -2.474565 -0.180376
H -0.996503 -2.504938 -0.185508
H -1.253274 3.061468 -0.049706
H 1.253274 3.061467 -0.049706
H 3.402666 1.843930 -0.078935
H 4.678831 -0.274421 -0.127122
H 3.465690 -2.474566 -0.180375
H 0.996502 -2.504938 -0.185507
He -2.019917 -0.362211 2.901031

He2Ph, I
E = -545.237029
C 0.000000 1.500242 -1.595728
C 0.000000 0.729325 -0.406860
C 0.000000 1.424346 0.840751
C 0.000000 2.841297 0.853996
C 0.000000 3.566096 -0.324223
C 0.000000 2.885284 -1.559314
C 0.000000 0.681100 2.070824
C -0.000000 -0.681100 2.070824
C -0.000000 -1.424346 0.840751
C -0.000000 -0.729325 -0.406860
C -0.000000 -1.500242 -1.595728
C -0.000000 -2.885284 -1.559314
C -0.000000 -3.566096 -0.324223
C -0.000000 -2.841297 0.853996
H 0.000000 -3.356136 1.815881
H 0.000000 -4.656121 -0.300317
H 0.000000 -3.451113 -2.491255
H 0.000000 -1.006197 -2.565006
H 0.000000 -1.233843 3.011103
H 0.000000 1.233843 3.011103
H 0.000000 3.356136 1.815881
H 0.000000 4.656121 -0.300317
H 0.000000 3.451113 -2.491255
H 0.000000 1.006197 -2.565006
He 3.027723 -0.000000 0.626460
He -3.027723 0.000000 0.626460

He2Ph-, I
E = -545.244095
C 0.000000 1.502910 -1.584539
C 0.000000 0.739973 -0.407074
C 0.000000 1.434956 0.866671
C 0.000000 2.872122 0.844715
C 0.000000 3.585492 -0.343175
C 0.000000 2.910547 -1.580360
C 0.000000 0.705235 2.072630
C -0.000000 -0.705235 2.072630
C -0.000000 -1.434956 0.866671
C -0.000000 -0.739973 -0.407074
C -0.000000 -1.502910 -1.584539
C -0.000000 -2.910547 -1.580360
C -0.000000 -3.585492 -0.343175
C -0.000000 -2.872122 0.844715
H -0.000000 -3.402603 1.800397
H -0.000000 -4.678997 -0.318473
H -0.000000 -3.465872 -2.519384
H -0.000000 -0.996702 -2.550089
H -0.000000 -1.253249 3.017720
H 0.000000 1.253249 3.017720
H 0.000000 3.402603 1.800397
H 0.000000 4.678997 -0.318473
H 0.000000 3.465872 -2.519384
H 0.000000 0.996702 -2.550089
He 3.017321 -0.000000 0.678310
He -3.017321 0.000000 0.678310

(H2)3Ph, II
E = -542.914196
C 3.562414 -0.275961 -0.071176
C 2.838536 0.899135 0.020356
C 1.421683 0.886714 0.019320
C 0.725647 -0.356572 -0.075962
C 1.495794 -1.542324 -0.167942
C 2.880646 -1.506936 -0.166667
C 0.679725 2.113400 0.112681
C -0.682727 2.113870 0.111105
C -1.425962 0.888229 0.016394
C -0.731584 -0.355675 -0.077613
C -2.842615 0.902472 0.015355
C -3.567911 -0.271790 -0.076997
C -2.887563 -1.503467 -0.171983
C -1.502644 -1.540415 -0.171473
H 0.390165 0.774195 -2.711081
H 0.015418 0.145957 2.853837
H 0.372654 -2.428800 2.466917
H -1.008356 -2.506438 -0.244260
H 1.000371 -2.507679 -0.241262
H 1.232886 3.050564 0.183322
H -1.234982 3.051713 0.180486
H -3.453865 -2.432068 -0.246722
H 3.353996 1.857758 0.094400
H -3.357019 1.861754 0.088758
H 3.445938 -2.436228 -0.240418
H -4.657876 -0.247534 -0.077382
H 4.652377 -0.253090 -0.070177
H -0.162786 0.877450 -3.221213
H 0.016766 0.904739 2.832900

H -0.386324 -2.416374 2.459516

(H2)3Ph-, II
E = -542.923886
C 3.608344 -0.394004 -0.000608
C 2.909151 0.801736 -0.004427
C 1.474042 0.839824 -0.026599
C 0.764188 -0.424040 -0.047225
C 1.512696 -1.610939 -0.039592
C 2.918502 -1.622949 -0.017752
C 0.758639 2.055821 -0.025442
C -0.652491 2.072097 -0.046534
C -1.395400 0.874002 -0.069662
C -0.715197 -0.406653 -0.076628
C -2.831745 0.868188 -0.089759
C -3.558158 -0.311016 -0.130678
C -2.897538 -1.554955 -0.148514
C -1.490844 -1.575613 -0.117066
H 0.152085 1.257781 -2.619862
H 0.465620 1.137193 3.294828
H -2.075522 -0.564544 3.232524
H -0.995181 -2.546258 -0.123998
H 0.994900 -2.570103 -0.052538
H 1.317533 2.993702 -0.010997
H -1.189457 3.022905 -0.042673
H -3.462880 -2.487216 -0.177057
H 3.450322 1.751004 0.010516
H -3.351323 1.829348 -0.075616
H 3.462701 -2.568331 -0.012535
H -4.650828 -0.273698 -0.146253
H 4.701697 -0.382549 0.016703
H 0.142718 0.941680 -3.313894
H 0.557858 1.391716 2.581374
H -1.945370 -0.601625 2.482397

(H2)3Ph, IV
E = -542.913895
C 3.563570 -0.216350 -0.183307
C 2.838320 0.956406 -0.069097
C 1.421860 0.943642 -0.080801
C 0.727459 -0.297106 -0.213479
C 1.498374 -1.479769 -0.331614
C 2.883390 -1.444522 -0.316696
C 0.677953 2.166721 0.046604
C -0.684229 2.165536 0.046456
C -1.426274 0.941260 -0.080209
C -0.730504 -0.298595 -0.212182
C -2.842864 0.951915 -0.066456
C -3.566091 -0.222301 -0.178918
C -2.884068 -1.449448 -0.311318
C -1.499398 -1.482530 -0.326621
H -1.649808 -0.572600 2.978969
H 1.704890 -0.447321 3.029051
H 0.330499 -2.533465 2.151517
H -1.003368 -2.445371 -0.422222
H 1.004206 -2.443297 -0.430216
H 1.229909 3.102263 0.145469
H -1.237635 3.100252 0.145110
H -3.448878 -2.377751 -0.399243
H 3.352819 1.912603 0.035320
H -3.358858 1.907436 0.037322
H 3.449407 -2.371842 -0.406973
H -4.655987 -0.200958 -0.165077
H 4.653415 -0.193330 -0.170177
H 2.190666 -0.497740 2.447881
H -0.250251 -3.020109 2.199061
H -2.176010 -0.327928 2.490031

(H2)3Ph-, IV
E = -542.923330
C 3.579257 -0.231370 -0.204852
C 2.862812 0.948836 -0.076106
C 1.425332 0.967561 -0.076707
C 0.734072 -0.301909 -0.203644
C 1.499961 -1.470562 -0.332394
C 2.908560 -1.462063 -0.337602
C 0.692332 2.165638 0.040791
C -0.717674 2.163676 0.035931
C -1.443448 0.961291 -0.080392
C -0.745122 -0.305337 -0.197458
C -2.880058 0.934909 -0.082515
C -3.589743 -0.249570 -0.191841
C -2.911012 -1.480235 -0.301940
C -1.504921 -1.480727 -0.299034
H -2.052914 -0.508246 3.128464
H 2.121083 -0.510647 3.092150
H 0.413975 -2.350074 2.088177
H -0.995679 -2.441019 -0.375835
H 0.996464 -2.431415 -0.435783
H 1.238140 3.107281 0.134415
H -1.267742 3.103158 0.125141
H -3.463617 -2.417312 -0.380085
H 3.390825 1.899872 0.026677
H -3.413057 1.884546 0.008341
H 3.465957 -2.394141 -0.437496
H -4.682978 -0.228356 -0.187328
H 4.672400 -0.204039 -0.199714
H 2.257345 -0.258407 2.386407
H -0.002067 -2.846981 2.487501
H -2.140215 -0.365047 2.385542

(H2)3Ph, I
E = -542.915490
C 2.840150 0.881466 -0.013653
C 1.423520 0.869144 -0.022980
C 0.728295 -0.376308 -0.083242
C 1.497903 -1.564151 -0.136203
C 2.882754 -1.529452 -0.125854
C 3.564333 -0.296274 -0.063188
C -0.729228 -0.375825 -0.083887

C -1.423660 0.869912 -0.023991
C -0.680766 2.098501 0.032980
C 0.681221 2.098229 0.033402
C -2.840229 0.883148 -0.016240
C -3.565166 -0.294096 -0.066746
C -2.884352 -1.527689 -0.128764
C -1.499393 -1.563217 -0.137730
H 2.178778 -0.426907 2.601978
H -2.161535 -0.455217 2.586942
H 0.398813 0.650640 -2.841593
H 3.355419 1.841642 0.035860
H 4.654200 -0.273559 -0.053570
H 3.447970 -2.460784 -0.165222
H 1.003095 -2.531730 -0.181966
H 1.234152 3.037276 0.077443
H -1.233219 3.037867 0.076383
H -3.354936 1.843654 0.033068
H -4.655026 -0.270678 -0.058048
H -3.450090 -2.458681 -0.168628
H -1.005139 -2.531137 -0.182754
H 1.628674 -0.351172 3.119802
H -1.675145 -0.347470 3.160154
H -0.338306 0.655933 -3.023282

(H2)3Ph-, I
E = -542.924374
C 2.871002 0.867470 0.014884
C 1.434551 0.890591 0.009011
C 0.739750 -0.378283 -0.094932
C 1.502272 -1.552767 -0.188327
C 2.908949 -1.549024 -0.184629
C 3.583869 -0.316392 -0.080132
C -0.739750 -0.378284 -0.094930
C -1.434552 0.890590 0.009011
C -0.705467 2.093473 0.103124
C 0.705465 2.093474 0.103124
C -2.871003 0.867468 0.014884
C -3.583868 -0.316395 -0.080131
C -2.908947 -1.549027 -0.184625
C -1.502271 -1.552768 -0.188323
H 1.803469 -0.589887 2.486115
H -1.803418 -0.589824 2.486128
H -0.000019 1.464009 -2.529056
H 3.401445 1.819146 0.098294
H 4.677029 -0.292652 -0.070366
H 3.463944 -2.485244 -0.255634
H 0.995520 -2.514757 -0.263819
H 1.253477 3.035131 0.178314
H -1.253480 3.035130 0.178313
H -3.401447 1.819143 0.098292
H -4.677029 -0.292656 -0.070365
H -3.463942 -2.485247 -0.255630
H -0.995518 -2.514759 -0.263814
H 1.915636 -0.631227 3.238900
H -1.915647 -0.631244 3.238900
H -0.000016 1.194180 -3.242620

(H2)3Ph, III
E = -542.913627
C -2.840950 0.852385 -0.213512
C -1.424405 0.838626 -0.222174
C -0.729222 -0.407375 -0.168589
C -1.499273 -1.594884 -0.112488
C -2.884269 -1.558363 -0.106086
C -3.565366 -0.324430 -0.156458
C 0.729134 -0.407364 -0.168638
C 1.424307 0.838644 -0.222252
C 0.681192 2.067366 -0.282299
C -0.681305 2.067355 -0.282268
C 2.840854 0.852414 -0.213661
C 3.565289 -0.324397 -0.156684
C 2.884208 -1.558341 -0.106307
C 1.499208 -1.594870 -0.112610
H -1.740668 -0.068547 2.935541
H 0.001357 1.883423 2.548363
H 2.210119 -0.533919 2.561372
H -3.355800 1.813328 -0.251854
H -4.655226 -0.300613 -0.149150
H -3.449890 -2.489300 -0.061354
H -1.004859 -2.562846 -0.069073
H -1.233849 3.006678 -0.324901
H 1.233723 3.006695 -0.324963
H 3.355692 1.813363 -0.251998
H 4.655149 -0.300569 -0.149428
H 3.449839 -2.489271 -0.061616
H 1.004804 -2.562837 -0.069179
H 1.742959 -0.071836 2.940819
H 0.000440 1.910110 3.307823
H -2.210187 -0.534451 2.563759

(H2)3Ph-, III
E = -542.923585
C -2.887788 0.857668 -0.179974
C -1.451425 0.878838 -0.196443
C -0.757389 -0.394303 -0.159388
C -1.521171 -1.571020 -0.111469
C -2.927701 -1.565668 -0.102626
C -3.601744 -0.328457 -0.136396
C 0.721934 -0.396705 -0.175753
C 1.417726 0.875150 -0.210388
C 0.688940 2.082078 -0.241604
C -0.721603 2.083912 -0.242070
C 2.854039 0.852187 -0.215208
C 3.566260 -0.335773 -0.197275
C 2.890092 -1.571990 -0.170184
C 1.482639 -1.575182 -0.161736
H -1.935786 -0.338573 3.185153
H 0.502057 1.902239 2.408423
H 2.341561 -0.793714 2.436963
H -3.417390 1.813268 -0.201701

H -4.694805 -0.302694 -0.123490
H -3.483228 -2.503347 -0.060886
H -1.015070 -2.535742 -0.077236
H -1.268597 3.028911 -0.265961
H 1.238044 3.025676 -0.272073
H 3.385018 1.807029 -0.234164
H 4.659412 -0.312366 -0.200367
H 3.444450 -2.511153 -0.157315
H 0.975524 -2.539670 -0.137516
H 2.114010 -0.489363 3.096815
H 0.467332 1.843072 3.169251
H -1.829385 -0.437980 2.437188

(H2O)3Ph, I
E = -768.720383
C 2.763146 -1.203446 0.419314
C 1.346870 -1.175716 0.413803
C 0.654528 -0.589631 -0.688623
C 1.426830 -0.063736 -1.754154
C 2.812872 -0.101748 -1.728580
C 3.490803 -0.672986 -0.630701
C -0.801807 -0.535344 -0.665204
C -1.500185 -1.066490 0.460523
C -0.762129 -1.667425 1.538506
C 0.600753 -1.720475 1.515955
C -2.913876 -0.986864 0.512212
C -3.632917 -0.401326 -0.513714
C -2.949873 0.118570 -1.633401
C -1.566974 0.048791 -1.705975
O -1.095361 2.779590 0.247256
O 0.077742 1.549144 2.490832
H 3.275783 -1.647098 1.273482
H 4.580219 -0.694464 -0.612431
H 3.380673 0.314428 -2.560615
H 0.935785 0.389729 -2.612077
H 1.149621 -2.175878 2.340844
H -1.316983 -2.079619 2.381926
H -3.430276 -1.391794 1.383258
H -4.719639 -0.339597 -0.459667
H -3.511275 0.578264 -2.446766
H -1.071365 0.461235 -2.581897
H -1.592279 2.151249 -0.291358
H -0.972980 2.330115 1.109126
H 0.059482 0.584534 2.444641
H 0.864799 1.808540 1.968599
O 1.722961 2.566277 0.446512
H 2.090488 1.904796 -0.153117
H 0.826962 2.752429 0.099491

(H2O)3Ph-, I
E = -768.748161
C 2.611259 -1.376506 -0.285920
C 1.184500 -1.290083 -0.158063
C 0.500197 -0.246400 -0.894079
C 1.262126 0.619378 -1.692654
C 2.663599 0.524869 -1.790423
C 3.328137 -0.492308 -1.074630
C -0.966678 -0.123489 -0.752117
C -1.665477 -1.064237 0.101632
C -0.950573 -2.067876 0.785807
C 0.454334 -2.169585 0.671955
C -3.088345 -0.921368 0.231321
C -3.785059 0.079344 -0.424200
C -3.106383 0.999049 -1.248686
C -1.712559 0.879683 -1.394055
O -0.681162 2.256536 1.529809
O 0.753444 0.182953 2.840222
H 3.133230 -2.155452 0.273750
H 4.415302 -0.580721 -1.137441
H 3.220493 1.220259 -2.418393
H 0.765281 1.412008 -2.250949
H 0.991482 -2.958698 1.201484
H -1.498607 -2.759357 1.428115
H -3.620011 -1.624777 0.875912
H -4.867179 0.160107 -0.294660
H -3.647232 1.795322 -1.760329
H -1.203140 1.598921 -2.034810
H -1.130818 1.855984 0.769666
H -0.435527 1.489165 2.087417
H 0.598686 -0.598924 2.279856
H 1.469445 0.663090 2.380918
O 2.160904 2.107309 1.209099
H 2.354037 1.658308 0.371584
H 1.214601 2.347555 1.139501

He3Ph, II
E = -548.146288
C -0.729262 -0.357758 -0.090830
C 0.729262 -0.357758 -0.090830
H -1.006024 -2.507607 -0.277374
C 0.681105 2.110519 0.125229
C -0.681105 2.110519 0.125230
H 1.006024 -2.507608 -0.277369
C -1.500179 -1.542010 -0.194424
C 1.424298 0.885124 0.018041
C -1.424298 0.885123 0.018042
C 1.500179 -1.542010 -0.194422
C -2.885151 -1.505801 -0.191185
C 2.841239 0.898316 0.019164
C -2.841239 0.898316 0.019166
C 2.885151 -1.505801 -0.191183
C -3.566006 -0.275423 -0.083722
C 3.566006 -0.275423 -0.083722
H 1.233863 3.047211 0.207151
H -1.233863 3.047211 0.207153
H -3.450968 -2.434200 -0.272305
H 3.356086 1.856528 0.103003
H -3.356086 1.856528 0.103006
H 3.450968 -2.434200 -0.272301
H -4.656031 -0.251637 -0.081737

H 4.656031 -0.251636 -0.081738
He 0.000001 -2.353972 2.705802
He -0.000001 0.438393 3.022041
He -0.000000 0.927485 -3.020242

He3Ph-, II
E = -548.153378
C -0.739880 -0.362549 -0.090436
C 0.739883 -0.362551 -0.090434
H -0.996661 -2.498399 -0.263953
C 0.705223 2.109005 0.110777
C -0.705216 2.109006 0.110777
H 0.996661 -2.498401 -0.263944
C -1.502888 -1.536019 -0.186423
C 1.434890 0.907013 0.013219
C -1.434885 0.907015 0.013218
C 1.502889 -1.536022 -0.186416
C -2.910441 -1.531845 -0.186252
C 2.872062 0.885117 0.011781
C -2.872057 0.885122 0.011779
C 2.910442 -1.531850 -0.186242
C -3.585404 -0.298799 -0.085296
C 3.585407 -0.298806 -0.085290
H 1.253261 3.050961 0.187454
H -1.253252 3.050963 0.187458
H -3.465781 -2.467711 -0.262877
H 3.402529 1.837608 0.089763
H -3.402522 1.837614 0.089763
H 3.465781 -2.467718 -0.262863
H -4.678906 -0.274200 -0.083281
H 4.678910 -0.274209 -0.083273
He -0.000075 -2.186236 2.807998
He -0.000014 0.546443 3.010900
He 0.000004 0.960025 -3.010308

He3Ph, IV
E = -548.146061
C -0.766723 -0.338470 -0.187904
C 0.691654 -0.328359 -0.218630
H -1.031125 -2.493887 -0.320152
C 0.629764 2.143583 -0.052592
C -0.732213 2.134232 -0.025302
H 0.979900 -2.479621 -0.366993
C -1.530841 -1.530179 -0.248258
C 1.379766 0.921260 -0.150105
C -1.468572 0.901659 -0.091516
C 1.468887 -1.509207 -0.314528
C -2.915668 -1.503538 -0.216054
C 2.796208 0.944243 -0.179931
C -2.885211 0.904993 -0.060256
C 2.853233 -1.463178 -0.342942
C -3.603177 -0.275822 -0.121376
C 3.527047 -0.226243 -0.275381
H 1.177107 3.085693 -0.001435
H -1.289925 3.068692 0.048527
H -3.476223 -2.437455 -0.264033
H 3.305646 1.907500 -0.126626
H -3.405304 1.861226 0.013568
H 3.424049 -2.389081 -0.417584
H -4.693058 -0.259787 -0.096336
H 4.616617 -0.195067 -0.297849
He 0.000811 -2.263319 2.631918
He 2.173037 -0.459896 2.794177
He -0.310154 0.564184 2.942688

He3Ph-, IV
E = -548.153233
C -0.780571 -0.340253 -0.184837
C 0.698966 -0.330390 -0.218505
H -1.025633 -2.480875 -0.308495
C 0.650925 2.144372 -0.063059
C -0.759232 2.135084 -0.032709
H 0.966976 -2.467347 -0.357167
C -1.537059 -1.520604 -0.239506
C 1.387028 0.945602 -0.154850
C -1.482248 0.926556 -0.091168
C 1.467951 -1.500308 -0.309716
C -2.944128 -1.525978 -0.207880
C 2.823827 0.933381 -0.190385
C -2.918885 0.894806 -0.060034
C 2.875164 -1.486477 -0.342836
C -3.625591 -0.295602 -0.116909
C 3.543168 -0.247363 -0.282048
H 1.193764 3.091389 -0.017063
H -1.312061 3.074724 0.038248
H -3.494295 -2.467000 -0.252576
H 3.349001 1.890773 -0.143342
H -3.454353 1.845084 0.010570
H 3.435316 -2.419906 -0.414387
H -4.718922 -0.278365 -0.090755
H 4.636165 -0.215245 -0.306555
He -0.034425 -2.149595 2.705556
He 2.249366 -0.490004 2.783377
He -0.200868 0.654504 2.915153

He3Ph, I
E = -548.146294
C -1.500131 -1.565823 -0.147831
C -0.729355 -0.378675 -0.082879
C -1.424449 0.866898 -0.013946
C -2.841281 0.879994 -0.011852
C -3.565922 -0.296507 -0.075779
C -2.885107 -1.529582 -0.144496
C -0.681133 2.095252 0.053242
C 0.681132 2.095252 0.053244
C 1.424449 0.866898 -0.013943
C 0.729355 -0.378675 -0.082879
C 1.500131 -1.565822 -0.147836
C 2.885107 -1.529582 -0.144500
C 3.565922 -0.296507 -0.075777

C 2.841281 0.879994 -0.011847
H 3.356134 1.840349 0.041838
H 4.655917 -0.272981 -0.073100
H 3.450930 -2.460168 -0.195030
H 1.005816 -2.533518 -0.201333
H 1.233766 3.034257 0.104351
H -1.233766 3.034257 0.104346
H -3.356135 1.840349 0.041830
H -4.655917 -0.272982 -0.073102
H -3.450930 -2.460168 -0.195021
H -1.005816 -2.533518 -0.201322
He 0.000001 0.807398 -3.062464
He 1.933622 -0.422341 2.963488
He -1.933623 -0.422336 2.963488

He3Ph-, I
E = -548.153471
C -1.502739 -1.550623 -0.156943
C -0.739988 -0.375337 -0.085262
C -1.434978 0.895910 -0.006615
C -2.872022 0.873808 -0.007371
C -3.585245 -0.311913 -0.078905
C -2.910280 -1.546657 -0.154718
C -0.705251 2.099693 0.067542
C 0.705267 2.099692 0.067542
C 1.434991 0.895907 -0.006616
C 0.739999 -0.375339 -0.085262
C 1.502746 -1.550627 -0.156942
C 2.910288 -1.546663 -0.154715
C 3.585256 -0.311921 -0.078902
C 2.872036 0.873801 -0.007370
H 3.402507 1.827599 0.051766
H 4.678717 -0.287394 -0.076067
H 3.465551 -2.484029 -0.211210
H 0.996478 -2.514277 -0.216182
H 1.253242 3.043087 0.125126
H -1.253224 3.043089 0.125126
H -3.402491 1.827607 0.051765
H -4.678706 -0.287384 -0.076070
H -3.465545 -2.484021 -0.211213
H -0.996472 -2.514274 -0.216185
He -0.000094 0.884679 -3.040399
He 2.020411 -0.489390 2.950326
He -2.020581 -0.489484 2.950251

He3Ph, III
E = -548.145950
C 1.500335 -1.575708 -0.165585
C 0.729465 -0.386814 -0.177605
C 1.424540 0.860736 -0.192631
C 2.841445 0.873771 -0.194077
C 3.566043 -0.304444 -0.182944
C 2.885294 -1.539310 -0.168810
C 0.681102 2.090825 -0.207888
C -0.681102 2.090825 -0.207888
C -1.424540 0.860736 -0.192631
C -0.729465 -0.386814 -0.177605
C -1.500335 -1.575708 -0.165585
C -2.885294 -1.539310 -0.168810
C -3.566043 -0.304444 -0.182944
C -2.841445 0.873771 -0.194077
He -2.180162 -0.395452 2.873319
He -0.000001 1.106704 2.914909
He 2.180161 -0.395451 2.873319
H -3.356335 1.835554 -0.205414
H -4.656034 -0.280791 -0.184832
H -3.451132 -2.471215 -0.159777
H -1.005954 -2.544791 -0.154356
H -1.233764 3.031108 -0.219935
H 1.233764 3.031108 -0.219935
H 3.356335 1.835554 -0.205414
H 4.656034 -0.280791 -0.184831
H 3.451133 -2.471215 -0.159777
H 1.005954 -2.544791 -0.154357

He3Ph-, III
E = -548.153158
C 1.502942 -1.559169 -0.173193
C 0.740087 -0.381679 -0.178803
C 1.435051 0.892133 -0.187310
C 2.872180 0.869845 -0.188613
C 3.585374 -0.318066 -0.183594
C 2.910469 -1.555041 -0.175710
C 0.705221 2.098120 -0.195507
C -0.705220 2.098120 -0.195507
C -1.435051 0.892133 -0.187310
C -0.740087 -0.381679 -0.178803
C -1.502942 -1.559169 -0.173193
C -2.910469 -1.555041 -0.175710
C -3.585374 -0.318066 -0.183594
C -2.872180 0.869845 -0.188613
He -2.233448 -0.430553 2.865922
He -0.000000 1.027651 2.885665
He 2.233447 -0.430552 2.865921
H -3.402696 1.825444 -0.194938
H -4.678833 -0.293382 -0.185207
H -3.465735 -2.494100 -0.171197
H -0.996598 -2.524589 -0.167211
H -1.253216 3.043227 -0.202576
H 1.253216 3.043227 -0.202575
H 3.402696 1.825443 -0.194937
H 4.678833 -0.293383 -0.185207
H 3.465735 -2.494100 -0.171197
H 0.996598 -2.524589 -0.167210

Ph
E = -539.418297
C 0.000000 1.500498 -1.569244
C 0.000000 0.729518 -0.380311
C 0.000000 1.424622 0.867497

C 0.000000 2.841599 0.880672
C 0.000000 3.566316 -0.297645
C 0.000000 2.885588 -1.532720
C 0.000000 0.681143 2.097782
C -0.000000 -0.681143 2.097782
C -0.000000 -1.424622 0.867497
C -0.000000 -0.729518 -0.380311
C -0.000000 -1.500498 -1.569244
C -0.000000 -2.885588 -1.532720
C -0.000000 -3.566316 -0.297645
C -0.000000 -2.841599 0.880672
H 0.000000 -3.356439 1.842559
H 0.000000 -4.656345 -0.273734
H 0.000000 -3.451485 -2.464681
H 0.000000 -1.006397 -2.538501
H 0.000000 -1.233864 3.038173
H 0.000000 1.233864 3.038173
H 0.000000 3.356439 1.842559
H 0.000000 4.656345 -0.273734
H 0.000000 3.451485 -2.464681
H 0.000000 1.006397 -2.538501

Ph-
E = -539.425261
C 0.000000 1.503121 -1.555822
C 0.000000 0.740127 -0.378316
C 0.000000 1.435201 0.895679
C 0.000000 2.872405 0.873575
C 0.000000 3.585728 -0.314412
C 0.000000 2.910858 -1.551582
C 0.000000 0.705316 2.101781
C -0.000000 -0.705316 2.101781
C -0.000000 -1.435201 0.895679
C -0.000000 -0.740127 -0.378316
C -0.000000 -1.503121 -1.555822
C -0.000000 -2.910858 -1.551582
C -0.000000 -3.585728 -0.314412
C -0.000000 -2.872405 0.873575
H -0.000000 -3.402912 1.829258
H -0.000000 -4.679248 -0.289695
H -0.000000 -3.466241 -2.490645
H -0.000000 -0.996855 -2.521358
H -0.000000 -1.253289 3.047012
H 0.000000 1.253289 3.047012
H 0.000000 3.402912 1.829258
H 0.000000 4.679248 -0.289695
H 0.000000 3.466241 -2.490645
H 0.000000 0.996855 -2.521358

wB97XD minima

Ph2
E = -1078.438423
C -1.849983 2.952948 -0.010816
C -0.494091 2.559286 0.041708
C -0.014370 1.571483 -0.853515
C -0.929124 1.001214 -1.767042
C -2.249496 1.398120 -1.802952
C -2.717210 2.386592 -0.919581
C 1.388698 1.180824 -0.794631
C 2.232955 1.783146 0.170847
C 1.698980 2.770097 1.070709
C 0.398721 3.145055 1.004709
C 3.595752 1.414458 0.237511
C 4.120254 0.480316 -0.629179
C 3.285572 -0.121072 -1.588026
C 1.951725 0.220103 -1.664389
H -2.201949 3.715770 0.684978
H -3.762666 2.692327 -0.946813
H -2.935726 0.929885 -2.507195
H -0.602191 0.222690 -2.452316
H 0.004531 3.899928 1.686193
H 2.368654 3.219065 1.805295
H 4.231584 1.889354 0.986001
H 5.173246 0.205606 -0.570838
H 3.690361 -0.870522 -2.267288
H 1.330280 -0.275189 -2.406467
C -1.063188 -0.087402 2.089479
C 0.239135 -0.454122 2.131222
C 0.757109 -1.450996 1.233600
C -0.098295 -2.051086 0.276144
C -1.502556 -1.658362 0.234088
C -1.972754 -0.683480 1.148391
C -2.424915 -2.209288 -0.683866
C -3.748772 -1.817977 -0.693132
C -4.211203 -0.854952 0.221364
C -3.331066 -0.298592 1.123897
C 0.454007 -3.015355 -0.597010
C 1.784286 -3.376243 -0.518015
C 2.625238 -2.782055 0.439136
C 2.115322 -1.831548 1.295927
H -0.171577 -3.495871 -1.346436
H 2.182605 -4.125982 -1.201511
H 3.676528 -3.063287 0.493314
H 2.761110 -1.348425 2.029321
H 0.924207 0.011844 2.839285
H -1.444848 0.678815 2.764453
H -3.672617 0.457926 1.830942
H -5.257149 -0.549047 0.211526
H -4.438955 -2.261886 -1.410461
H -2.099822 -2.959657 -1.401456

Ph2-
E = -1078.441141
C 2.129473 -3.324389 -0.710303
C 2.425078 -1.944936 -0.457940
C 1.745760 -1.304690 0.642045
C 0.861761 -2.058380 1.415937
C 0.589313 -3.411006 1.152827
C 1.233536 -4.031181 0.073359

C 1.972078 0.139396 0.858926
C 2.928848 0.828437 0.028681
C 3.587652 0.146007 -1.010245
C 3.323695 -1.215420 -1.258517
C 3.149739 2.220858 0.281708
C 2.443292 2.904079 1.248140
C 1.474287 2.240654 2.023463
C 1.263802 0.877001 1.815474
H 2.629222 -3.815842 -1.548112
H 1.031951 -5.081597 -0.152100
H -0.117061 -3.961216 1.774478
H 0.338042 -1.586366 2.246557
H 3.825406 -1.728461 -2.081715
H 4.304003 0.690197 -1.628965
H 3.890013 2.744396 -0.328066
H 2.626935 3.970269 1.401121
H 0.887143 2.782455 2.764602
H 0.503881 0.380383 2.418165
C -0.794470 -1.028201 -1.258828
C -0.175136 0.156159 -1.479130
C -0.773810 1.391564 -1.057232
C -2.045528 1.390368 -0.430185
C -2.716946 0.115501 -0.200543
C -2.072925 -1.081642 -0.605911
C -3.976874 0.011934 0.432853
C -4.566104 -1.215639 0.667779
C -3.911826 -2.400703 0.283498
C -2.684464 -2.328587 -0.341556
C -2.592378 2.630678 -0.029212
C -1.906116 3.813367 -0.223572
C -0.632563 3.803683 -0.821931
C -0.081059 2.610296 -1.232568
H -3.568055 2.667493 0.452808
H -2.347106 4.755490 0.104742
H -0.075633 4.733408 -0.940020
H 0.920755 2.578889 -1.660352
H 0.821619 0.182346 -1.920799
H -0.301640 -1.965032 -1.518805
H -2.149523 -3.235054 -0.626234
H -4.370182 -3.369394 0.486235
H -5.537496 -1.264726 1.161721
H -4.500958 0.911102 0.752630

H2
E = -1.161827
H 0.000000 0.000000 0.379041
H 0.000000 -0.000000 -0.379041

H2O
E = -76.398066
O 0.000000 0.000000 0.117242
H -0.000000 0.760984 -0.468968
H -0.000000 -0.760984 -0.468968

H2Ph, I
E = -540.370957
C -0.729366 -0.392090 -0.057106
C 0.729367 -0.392090 -0.057106
H -0.999743 -2.548521 -0.003721
C 0.677528 2.077517 -0.113273
C -0.677528 2.077516 -0.113273
H 0.999743 -2.548521 -0.003720
C -1.495009 -1.580051 -0.026076
C 1.419911 0.845474 -0.085188
C -1.419911 0.845474 -0.085188
C 1.495009 -1.580051 -0.026076
C -2.874728 -1.544796 -0.023177
C 2.833154 0.860344 -0.080946
C -2.833155 0.860344 -0.080946
C 2.874728 -1.544796 -0.023177
C -3.554663 -0.313468 -0.050525
C 3.554663 -0.313469 -0.050525
H 1.231343 3.016700 -0.133118
H -1.231342 3.016700 -0.133118
H -3.439611 -2.476540 0.001088
H 3.347633 1.821976 -0.101719
H -3.347633 1.821976 -0.101718
H 3.439611 -2.476540 0.001088
H -4.644227 -0.290173 -0.047395
H 4.644227 -0.290173 -0.047395
H -0.000003 0.968117 2.586472
H 0.000003 0.549847 3.218746

H2Ph-, I
E = -540.373543
C -0.740442 -0.398747 -0.047831
C 0.740442 -0.398747 -0.047831
H -0.990142 -2.536759 0.028283
C 0.705010 2.071851 -0.133974
C -0.705010 2.071851 -0.133974
H 0.990142 -2.536759 0.028283
C -1.497801 -1.572478 -0.006007
C 1.431055 0.868645 -0.092826
C -1.431055 0.868645 -0.092826
C 1.497801 -1.572478 -0.006007
C -2.899755 -1.570886 -0.006185
C 2.864307 0.845382 -0.091039
C -2.864307 0.845382 -0.091039
C 2.899755 -1.570886 -0.006185
C -3.573461 -0.338834 -0.049042
C 3.573461 -0.338834 -0.049042
H 1.253968 3.015457 -0.164854
H -1.253968 3.015457 -0.164854
H -3.453428 -2.509910 0.027407
H 3.395619 1.799688 -0.123425
H -3.395619 1.799688 -0.123425
H 3.453428 -2.509910 0.027407
H -4.666519 -0.314563 -0.049111
H 4.666519 -0.314563 -0.049111
H 0.000003 1.251655 2.487526

H 0.000001 0.981317 3.198716

H2Ph, II
E = -540.370743
C -3.513507 -0.281462 -0.139911
C -2.788468 0.890796 -0.118409
C -1.375646 0.871923 -0.091768
C -0.689113 -0.368128 -0.087689
C -1.457721 -1.554182 -0.112183
C -2.837362 -1.515126 -0.137122
C -0.630078 2.102057 -0.062106
C 0.724067 2.097849 -0.029085
C 1.463026 0.863148 -0.022584
C 0.769372 -0.372752 -0.051897
C 2.875840 0.873402 0.012888
C 3.593929 -0.303113 0.019351
C 2.910884 -1.532530 -0.009104
C 1.531339 -1.563421 -0.043946
H 1.033621 -2.530646 -0.065530
H -0.965243 -2.524238 -0.108012
H -1.181249 3.043014 -0.065966
H 1.280500 3.035474 -0.006180
H 3.472903 -2.466287 -0.003812
H -3.300221 1.854020 -0.118526
H 3.392946 1.833614 0.034909
H -3.404773 -2.445429 -0.153068
H 4.683229 -0.283107 0.046785
H -4.602785 -0.254877 -0.158023
H -2.094428 -0.306087 2.579485
H -1.773863 -0.206212 3.259322

H2Ph-, II
E = -540.373234
C -3.532136 -0.294775 -0.142533
C -2.819023 0.887537 -0.113229
C -1.385249 0.906153 -0.085672
C -0.699902 -0.365583 -0.084779
C -1.461425 -1.537075 -0.115612
C -2.863658 -1.530314 -0.145305
C -0.654917 2.106191 -0.055583
C 0.754018 2.101086 -0.024718
C 1.475331 0.894544 -0.020372
C 0.780281 -0.371018 -0.049355
C 2.908196 0.865344 0.012426
C 3.612723 -0.322217 0.016759
C 2.934517 -1.552530 -0.010523
C 1.533155 -1.548558 -0.042839
H 1.021956 -2.511479 -0.063727
H -0.957078 -2.503700 -0.113648
H -1.200384 3.052471 -0.056306
H 1.306200 3.043168 -0.001785
H 3.484587 -2.494237 -0.006437
H -3.347130 1.844039 -0.108444
H 3.442959 1.818073 0.034219
H -3.420642 -2.467559 -0.165549
H 4.705581 -0.302461 0.042252
H -4.624830 -0.266229 -0.161007
H -1.910334 -0.310729 2.506341
H -1.992363 -0.334062 3.262103

H2OPh, I
E = -615.611730
C -2.623211 0.905759 -0.564795
C -1.215963 0.882953 -0.430991
C -0.534223 -0.359857 -0.385364
C -1.301224 -1.544183 -0.480350
C -2.674797 -1.501444 -0.608551
C -3.346843 -0.265087 -0.650116
C 0.916264 -0.369367 -0.224989
C 1.606132 0.864731 -0.119451
C 0.872338 2.101427 -0.173500
C -0.473998 2.110788 -0.322662
C 3.010460 0.870983 0.040265
C 3.722722 -0.307567 0.095386
C 3.043086 -1.534996 -0.007534
C 1.672140 -1.562597 -0.163878
H -3.131872 1.869903 -0.591984
H -4.431606 -0.235508 -0.748594
H -3.241339 -2.429841 -0.673493
H -0.812933 -2.515388 -0.444494
H -1.021499 3.052884 -0.357788
H 1.426093 3.036929 -0.088870
H 3.525113 1.829322 0.119792
H 4.805278 -0.290969 0.219119
H 3.601125 -2.469955 0.036627
H 1.177101 -2.528355 -0.239483
H -0.950066 0.044253 2.423496
O -1.825247 -0.111358 2.789244
H -2.380713 -0.221669 2.010905

H2OPh-, I
E = -615.622056
C -2.691954 0.264477 -0.704495
C -1.275740 0.459389 -0.596257
C -0.475028 -0.663047 -0.169706
C -1.115089 -1.869321 0.123173
C -2.504710 -2.036891 0.014636
C -3.284403 -0.950034 -0.405167
C 0.989872 -0.480828 -0.053479
C 1.551422 0.819147 -0.330421
C 0.720115 1.884615 -0.720323
C -0.673003 1.705401 -0.855808
C 2.968986 0.973834 -0.198387
C 3.784171 -0.075833 0.172715
C 3.235946 -1.343712 0.434454
C 1.852722 -1.517535 0.315850
H -3.305880 1.107902 -1.028159
H -4.367995 -1.059147 -0.498085
H -2.965611 -2.995004 0.256320
H -0.523802 -2.723294 0.454001

H -1.303737 2.533651 -1.185850
H 1.171536 2.856917 -0.927130
H 3.401456 1.956280 -0.401956
H 4.862101 0.081373 0.262305
H 3.874296 -2.178461 0.725718
H 1.444179 -2.506823 0.523106
H -1.409114 2.163435 1.397330
O -2.103541 2.059978 2.064008
H -2.548931 1.265374 1.749631

HeH2Ph, II
E = -543.274154
C 3.556370 -0.285190 -0.129261
C 2.834943 0.889045 -0.106382
C 1.421813 0.874771 -0.112104
C 0.731318 -0.362756 -0.141824
C 1.496597 -1.550979 -0.165152
C 2.876419 -1.516265 -0.158856
C 0.679386 2.107033 -0.085384
C -0.675173 2.107341 -0.086125
C -1.417604 0.875276 -0.113631
C -0.727514 -0.362440 -0.142939
C -2.830685 0.889807 -0.104734
C -3.552542 -0.284479 -0.123487
C -2.872857 -1.515825 -0.154189
C -1.492924 -1.550583 -0.164192
He 1.793730 -0.311436 3.098399
H -0.997635 -2.518966 -0.185345
H 1.001277 -2.519373 -0.188009
H 1.233459 3.046082 -0.063584
H -1.229024 3.046479 -0.064675
H -3.437697 -2.447711 -0.168494
H 3.349598 1.850523 -0.083092
H -3.345160 1.851245 -0.079421
H 3.441103 -2.448249 -0.176769
H -4.642007 -0.261236 -0.113924
H 4.645928 -0.262140 -0.124013
H -2.066583 -0.347804 2.559889
H -1.706005 -0.254513 3.220194

HeH2Ph-, II
E = -543.276622
C 3.573570 -0.302931 -0.135962
C 2.865432 0.882131 -0.110184
C 1.432196 0.906826 -0.110239
C 0.740672 -0.360494 -0.140532
C 1.497075 -1.535384 -0.166039
C 2.898749 -1.535099 -0.163823
C 0.707260 2.110806 -0.080429
C -0.702012 2.111468 -0.077411
C -1.429046 0.909401 -0.105828
C -0.739865 -0.359799 -0.139695
C -2.862974 0.886119 -0.097325
C -3.572826 -0.298206 -0.124510
C -2.900710 -1.531222 -0.161241
C -1.498169 -1.533452 -0.167284
He 1.868745 -0.326426 3.109093
H -0.990969 -2.498258 -0.191390
H 0.988550 -2.499659 -0.187663
H 1.256866 3.054392 -0.057780
H -1.250306 3.055748 -0.051703
H -3.455044 -2.470058 -0.180222
H 3.397509 1.836312 -0.087682
H -3.393827 1.840596 -0.066083
H 3.451690 -2.474906 -0.183600
H -4.665718 -0.273276 -0.114739
H 4.666630 -0.279811 -0.133848
H -1.874001 -0.353736 2.480730
H -1.924983 -0.385463 3.238798

HeH2Ph, III
E = -543.274000
C 3.605682 -0.338866 -0.000113
C 2.897412 0.843036 -0.030163
C 1.485207 0.843459 -0.083668
C 0.781803 -0.386880 -0.106422
C 1.533864 -1.583412 -0.074954
C 2.912956 -1.563096 -0.023184
C 0.756774 2.083726 -0.108613
C -0.597443 2.098569 -0.152980
C -1.352773 0.874252 -0.179600
C -0.676030 -0.371121 -0.157114
C -2.765035 0.904272 -0.228623
C -3.498653 -0.261976 -0.257277
C -2.832514 -1.500843 -0.235383
C -1.454220 -1.551090 -0.185316
H -0.969888 -2.525186 -0.168885
H 1.028495 -2.546766 -0.091040
H 1.320448 3.016999 -0.088268
H -1.140762 3.043916 -0.169860
H -3.407084 -2.426686 -0.257000
H 3.421899 1.799291 -0.011808
H -3.268733 1.871686 -0.244747
H 3.467258 -2.501187 0.000503
H -4.587195 -0.226893 -0.295991
H 4.694640 -0.327382 0.041589
H 0.019206 0.947142 2.548648
H 0.208108 0.544627 3.162514
He -2.784281 -0.604868 3.257396

HeH2Ph-, III
E = -543.276600
C 3.615765 -0.363555 -0.019977
C 2.918586 0.827515 -0.068062
C 1.486290 0.864009 -0.107886
C 0.783499 -0.397318 -0.095374
C 1.528903 -1.578423 -0.045666
C 2.930201 -1.589750 -0.008497
C 0.772148 2.074385 -0.151889
C -0.637291 2.087488 -0.187360

C -1.374727 0.890601 -0.181432
C -0.696775 -0.383666 -0.137265
C -2.807616 0.880216 -0.221749
C -3.527780 -0.298122 -0.224002
C -2.866297 -1.536695 -0.183819
C -1.465064 -1.551008 -0.140196
H -0.966802 -2.520273 -0.108536
H 1.011855 -2.538243 -0.035246
H 1.330227 3.013098 -0.157245
H -1.176796 3.036468 -0.219796
H -3.428679 -2.471112 -0.184910
H 3.459120 1.777135 -0.075355
H -3.329478 1.839732 -0.253575
H 3.474341 -2.534118 0.030307
H -4.620047 -0.263860 -0.256893
H 4.708584 -0.349345 0.009609
H 0.278745 1.326644 2.453547
H 0.237880 1.086487 3.174509
He -2.468999 -0.478339 3.131317

HeH2Ph, I
E = -543.274025
C -3.554614 -0.325762 -0.004547
C -2.833066 0.848409 -0.003898
C -1.419827 0.833597 -0.001800
C -0.729316 -0.404262 -0.001244
C -1.494956 -1.592594 -0.001934
C -2.874656 -1.557408 -0.003253
C -0.677506 2.065894 -0.003215
C 0.677507 2.065894 -0.003215
C 1.419827 0.833597 -0.001800
C 0.729316 -0.404262 -0.001244
C 2.833066 0.848409 -0.003898
C 3.554614 -0.325762 -0.004547
C 2.874656 -1.557408 -0.003254
C 1.494956 -1.592594 -0.001934
H 0.999735 -2.561330 -0.001655
H -0.999735 -2.561330 -0.001655
H -1.231320 3.005238 -0.005166
H 1.231320 3.005238 -0.005166
H 3.439548 -2.489420 -0.003896
H -3.347537 1.810259 -0.005471
H 3.347537 1.810259 -0.005471
H -3.439548 -2.489420 -0.003896
H 4.644183 -0.302545 -0.006206
H -4.644183 -0.302545 -0.006206
H 0.000003 0.888003 -2.678734
H -0.000005 0.488492 -3.322890
He 0.000000 0.642307 3.142560

HeH2Ph-, I
E = -543.276659
C -3.573382 -0.349947 -0.007161
C -2.864238 0.834915 0.007374
C -1.430993 0.858182 0.006508
C -0.740391 -0.409819 -0.011193
C -1.497721 -1.584199 -0.025446
C -2.899638 -1.582645 -0.023559
C -0.704982 2.061959 0.020914
C 0.704982 2.061959 0.020914
C 1.430993 0.858182 0.006508
C 0.740390 -0.409819 -0.011193
C 2.864237 0.834915 0.007373
C 3.573382 -0.349947 -0.007161
C 2.899637 -1.582645 -0.023558
C 1.497720 -1.584199 -0.025445
H 0.990083 -2.548987 -0.038845
H -0.990083 -2.548987 -0.038846
H -1.253948 3.005959 0.031767
H 1.253948 3.005959 0.031767
H 3.453291 -2.522160 -0.035355
H -3.395569 1.789671 0.019411
H 3.395568 1.789671 0.019409
H -3.453292 -2.522160 -0.035356
H 4.666434 -0.325725 -0.005975
H -4.666435 -0.325724 -0.005975
H 0.000005 1.177397 -2.588848
H 0.000000 0.907469 -3.300004
He 0.000004 0.588136 3.168802

HePh, I
E = -542.111470
C -0.389102 -0.059990 -0.729532
C -0.389102 -0.059990 0.729532
H -2.545730 -0.012179 -1.000005
C 2.080845 -0.113757 0.677318
C 2.080845 -0.113757 -0.677318
H -2.545730 -0.012179 1.000005
C -1.577171 -0.033215 -1.495246
C 0.848709 -0.086808 1.419911
C 0.848709 -0.086808 -1.419911
C -1.577171 -0.033215 1.495246
C -1.542013 -0.033194 -2.875105
C 0.863302 -0.086215 2.833171
C 0.863302 -0.086215 -2.833171
C -1.542013 -0.033194 2.875105
C -0.310680 -0.059900 -3.554862
C -0.310680 -0.059900 3.554862
H 3.020009 -0.133970 1.231253
H 3.020009 -0.133970 -1.231253
H -2.473835 -0.012340 -3.440002
H 1.824942 -0.107081 3.347706
H 1.824942 -0.107081 -3.347706
H -2.473835 -0.012340 3.440002
H -0.287329 -0.059700 -4.644446
H -0.287329 -0.059700 4.644446
He 0.618597 3.163736 -0.000000

HePh-, I
E = -542.113058

C 0.388829 -0.059880 0.740395
C 0.388829 -0.059880 -0.740395
H 2.527585 -0.011116 0.990284
C -2.082623 -0.115100 -0.704706
C -2.082623 -0.115100 0.704706
H 2.527585 -0.011116 -0.990284
C 1.562830 -0.032720 1.497901
C -0.879565 -0.088181 -1.430885
C -0.879565 -0.088181 1.430885
C 1.562830 -0.032720 -1.497901
C 1.561363 -0.032170 2.900239
C -0.856033 -0.086928 -2.864536
C -0.856033 -0.086928 2.864536
C 1.561363 -0.032170 -2.900239
C 0.328675 -0.059734 3.573738
C 0.328675 -0.059734 -3.573738
H -3.026711 -0.135950 -1.253686
H -3.026711 -0.135950 1.253686
H 2.500737 -0.010614 3.453966
H -1.810661 -0.108197 -3.395995
H -1.810661 -0.108197 3.395995
H 2.500737 -0.010614 -3.453966
H 0.304321 -0.059703 4.666855
H 0.304321 -0.059703 -4.666855
He -0.636133 3.173851 0.000000

HePh, II
E = -542.111421
C -1.459802 -1.555880 -0.114918
C -0.691696 -0.369397 -0.088697
C -1.379036 0.870314 -0.094189
C -2.791864 0.888353 -0.124956
C -3.516003 -0.284140 -0.149557
C -2.839224 -1.517364 -0.144523
C -0.633876 2.100889 -0.066509
C 0.720388 2.097622 -0.034384
C 1.460051 0.863374 -0.027062
C 0.767013 -0.373014 -0.054298
C 1.529859 -1.563245 -0.046140
C 2.909410 -1.531493 -0.012784
C 3.591757 -0.301606 0.014011
C 2.872956 0.874439 0.006873
H 3.389519 1.834992 0.027382
H 4.681076 -0.280949 0.040308
H 3.472016 -2.464911 -0.007328
H 1.032602 -2.530770 -0.066554
H 1.276265 3.035630 -0.013328
H -1.185520 3.041623 -0.071727
H -3.304080 1.851424 -0.128306
H -4.605266 -0.258180 -0.172752
H -3.406128 -2.447996 -0.163745
H -0.966823 -2.525785 -0.111473
He -1.811627 -0.224099 3.145160

HePh-, II
E = -542.113013
C -1.459910 -1.540580 -0.117214
C -0.699822 -0.368344 -0.089548
C -1.386920 0.902142 -0.095209
C -2.820154 0.882395 -0.129955
C -3.532070 -0.300672 -0.155799
C -2.861850 -1.535434 -0.149641
C -0.657976 2.103506 -0.066276
C 0.751020 2.099774 -0.031958
C 1.474027 0.894475 -0.024494
C 0.780545 -0.372357 -0.052628
C 1.534890 -1.548725 -0.043777
C 2.936796 -1.551052 -0.009118
C 3.613177 -0.319939 0.017962
C 2.907187 0.866991 0.010640
H 3.440889 1.820364 0.031857
H 4.706018 -0.298583 0.045147
H 3.487990 -2.492141 -0.003441
H 1.024985 -2.512281 -0.064758
H 1.302147 3.042597 -0.010094
H -1.204439 3.049290 -0.071532
H -3.349080 1.838624 -0.134457
H -4.624803 -0.273401 -0.181203
H -3.417754 -2.473536 -0.169932
H -0.954750 -2.506847 -0.113015
He -1.942429 -0.233586 3.146756

(H2)2Ph, II
E = -541.533942
C 3.554708 -0.284821 -0.118287
C 2.833028 0.889600 -0.099878
C 1.420033 0.875252 -0.109713
C 0.729788 -0.362254 -0.139506
C 1.494876 -1.550493 -0.159659
C 2.874836 -1.516047 -0.148901
C 0.677745 2.107433 -0.082600
C -0.676740 2.107471 -0.082677
C -1.419041 0.875300 -0.109961
C -0.728883 -0.362241 -0.139688
C -2.832070 0.889598 -0.100257
C -3.553692 -0.284949 -0.119199
C -2.873796 -1.516110 -0.150238
C -1.493923 -1.550445 -0.160440
H -0.998506 -2.518716 -0.181044
H 0.999522 -2.518809 -0.180425
H 1.231756 3.046460 -0.060674
H -1.230717 3.046519 -0.060744
H -3.438407 -2.448116 -0.164293
H 3.347623 1.850954 -0.074348
H -3.346752 1.850917 -0.074810
H 3.439466 -2.448052 -0.162690
H -4.643156 -0.261851 -0.109337
H 4.644168 -0.261720 -0.108188
H 2.103026 -0.377653 2.563676
H 1.717744 -0.241761 3.202095

H -2.146752 -0.318087 2.555222
H -1.720231 -0.303851 3.181586

(H2)2Ph-, II
E = -541.536999
C 3.572818 -0.299145 -0.124560
C 2.863963 0.885527 -0.095777
C 1.430374 0.909902 -0.102078
C 0.740196 -0.358386 -0.136103
C 1.497427 -1.532838 -0.164598
C 2.899402 -1.531671 -0.160398
C 0.704554 2.112846 -0.071549
C -0.704554 2.112845 -0.071549
C -1.430374 0.909902 -0.102078
C -0.740196 -0.358386 -0.136103
C -2.863963 0.885526 -0.095777
C -3.572818 -0.299145 -0.124560
C -2.899402 -1.531671 -0.160398
C -1.497427 -1.532838 -0.164598
H -0.989467 -2.497198 -0.187615
H 0.989467 -2.497198 -0.187615
H 1.253549 3.056612 -0.045389
H -1.253549 3.056612 -0.045389
H -3.452912 -2.470942 -0.179852
H 3.395513 1.839585 -0.064831
H -3.395513 1.839585 -0.064831
H 3.452913 -2.470941 -0.179852
H -4.665682 -0.275174 -0.116238
H 4.665682 -0.275174 -0.116238
H 1.892227 -0.367804 2.483392
H 1.946060 -0.402489 3.240908
H -1.892227 -0.367793 2.483392
H -1.946061 -0.402489 3.240907

(H2)2Ph, I
E = -541.533736
C -3.591206 -0.324198 0.074851
C -2.874115 0.852364 0.106132
C -1.461337 0.844046 0.071806
C -0.766584 -0.389506 0.003865
C -1.527526 -1.580338 -0.026703
C -2.906999 -1.551587 0.008306
C -0.723799 2.078966 0.100788
C 0.630676 2.085506 0.064116
C 1.377023 0.857630 -0.003976
C 0.691404 -0.382371 -0.034750
C 2.789617 0.879022 -0.037818
C 3.515447 -0.291145 -0.099375
C 2.840246 -1.525035 -0.130322
C 1.460899 -1.566280 -0.099214
H 0.969367 -2.536505 -0.122157
H -1.029103 -2.545986 -0.078871
H -1.280904 3.014990 0.151977
H 1.180801 3.026753 0.085538
H 3.408361 -2.453805 -0.177928
H -3.391903 1.811061 0.157234
H 3.300419 1.842351 -0.011782
H -3.468295 -2.485448 -0.016541
H 4.604556 -0.262958 -0.123044
H -4.680537 -0.305967 0.101606
H -0.126898 1.092386 -2.645684
H -0.159414 0.628237 -3.244215
H 2.130721 -0.420967 2.626178
H 1.820352 -0.326576 3.311460

(H2)2Ph-, I
E = -541.537071
C -3.609773 -0.350408 0.068222
C -2.906162 0.836704 0.114318
C -1.473795 0.868115 0.081116
C -0.777763 -0.393878 -0.003375
C -1.529590 -1.571309 -0.047344
C -2.930484 -1.577668 -0.012904
C -0.753701 2.075003 0.125563
C 0.655745 2.082677 0.091437
C 1.386860 0.885023 0.010954
C 0.702358 -0.385622 -0.040216
C 2.820154 0.869210 -0.020853
C 3.534110 -0.310154 -0.100665
C 2.866434 -1.544960 -0.152582
C 1.464653 -1.554269 -0.120818
H 0.961101 -2.520505 -0.157893
H -1.017751 -2.531915 -0.111019
H -1.306616 3.014649 0.187334
H 1.200351 3.028525 0.126990
H 3.424049 -2.480055 -0.212782
H -3.441443 1.787230 0.176884
H 3.347285 1.825219 0.021450
H -3.479813 -2.519065 -0.049330
H 4.626666 -0.279961 -0.120725
H -4.702553 -0.332715 0.095642
H -0.106830 1.321905 -2.527159
H -0.126222 1.057685 -3.240324
H 1.924378 -0.445121 2.549600
H 2.003120 -0.496658 3.304214

(H2O)2Ph, II
E = -692.015455
C -1.564424 -0.853901 -1.390806
C -0.786716 -0.215134 -0.398311
C -1.462016 0.511411 0.615171
C -2.873762 0.579802 0.607194
C -3.606877 -0.053047 -0.373089
C -2.942138 -0.776081 -1.380120
C -0.706312 1.170365 1.648702
C 0.646789 1.100234 1.679491
C 1.374602 0.370673 0.674167
C 0.671489 -0.282511 -0.371405
C 1.424371 -0.976318 -1.347174
C 2.802856 -1.022166 -1.289251

C 3.493975 -0.375541 -0.248310
C 2.786232 0.309131 0.715959
O 0.627650 3.014672 -1.304480
H 3.309559 0.818722 1.525314
H 4.581878 -0.415732 -0.207341
H 3.357575 -1.564275 -2.054370
H 0.920570 -1.489313 -2.163032
H 1.209529 1.596697 2.469855
H -1.248973 1.722302 2.416311
H -3.376560 1.143051 1.393950
H -4.694873 0.004622 -0.369913
H -3.517510 -1.278809 -2.156809
H -1.081662 -1.422578 -2.182297
H 0.189625 2.803986 -0.474051
H 1.117251 2.214612 -1.515332
H -0.867617 -2.120957 1.569746
O -0.139127 -2.611330 1.962018
H 0.644617 -2.160564 1.634355

(H2O)2Ph-, II
E = -692.034960
C -1.497929 -1.725063 0.021063
C -0.740051 -0.550753 -0.026432
C -1.426504 0.712850 -0.136035
C -2.853207 0.687647 -0.238693
C -3.562533 -0.496573 -0.209748
C -2.894276 -1.724047 -0.070263
C -0.699558 1.920263 -0.103960
C 0.699559 1.920263 0.103953
C 1.426506 0.712852 0.136033
C 0.740054 -0.550753 0.026435
C 1.497933 -1.725062 -0.021057
C 2.894280 -1.724044 0.070268
C 3.562536 -0.496570 0.209750
C 2.853209 0.687650 0.238691
O 0.462797 0.852662 -3.095922
H 3.380937 1.638984 0.332362
H 4.652199 -0.474933 0.286450
H 3.450455 -2.660736 0.029849
H 0.992301 -2.684383 -0.131411
H 1.246266 2.863093 0.152017
H -1.246266 2.863092 -0.152026
H -3.380936 1.638981 -0.332366
H -4.652196 -0.474937 -0.286448
H -3.450450 -2.660739 -0.029841
H -0.992296 -2.684384 0.131420
H -0.017598 1.330171 -2.405096
H 0.975263 0.220485 -2.580698
H -0.975276 0.220497 2.580692
O -0.462810 0.852673 3.095918
H 0.017589 1.330180 2.405093

(H2O)2Ph, I
E = -692.019569
C 1.378861 -1.452736 -0.800680
C 0.555903 -0.313029 -0.654334
C 1.176264 0.961361 -0.648572
C 2.579627 1.060877 -0.777284
C 3.359281 -0.067060 -0.916535
C 2.748889 -1.334880 -0.930544
C 0.372075 2.147292 -0.508406
C -0.976382 2.068983 -0.386162
C -1.647593 0.794809 -0.373462
C -0.892634 -0.399653 -0.498214
C -1.584785 -1.631352 -0.445273
C -2.954940 -1.677326 -0.281082
C -3.698687 -0.489074 -0.164269
C -3.049735 0.726252 -0.210443
H -3.613333 1.655076 -0.115707
H -4.779753 -0.531100 -0.035161
H -3.461854 -2.641029 -0.240747
H -1.039643 -2.568751 -0.529025
H -1.577309 2.973714 -0.289637
H 0.871858 3.116381 -0.510932
H 3.040122 2.049063 -0.760866
H 4.441171 0.019327 -1.009273
H 3.360143 -2.230366 -1.036795
H 0.940836 -2.448055 -0.805699
H 2.367520 -0.896117 1.662990
O 2.196813 -0.852770 2.607738
H 1.373648 -0.345983 2.681857
H -0.233910 1.321669 2.017429
O -0.311315 0.619477 2.672668
H -0.970333 0.025742 2.299875

(H2O)2Ph-, I
E = -692.038275
C -1.306076 -1.477830 0.774672
C -0.499444 -0.345396 0.635300
C -1.118895 0.955589 0.687805
C -2.536746 1.011931 0.867910
C -3.300235 -0.131987 0.990891
C -2.694829 -1.398807 0.946053
C -0.336794 2.121828 0.555628
C 1.067510 2.044591 0.422395
C 1.720665 0.802409 0.342685
C 0.963269 -0.425896 0.414300
C 1.640382 -1.640527 0.266122
C 3.025908 -1.715283 0.078128
C 3.767534 -0.522343 0.029915
C 3.137839 0.699291 0.155503
H 3.719950 1.622165 0.106536
H 4.850023 -0.559309 -0.115112
H 3.515932 -2.682583 -0.033822
H 1.078882 -2.574032 0.298605
H 1.661731 2.956884 0.346659
H -0.830389 3.093617 0.612759
H -3.014971 1.993137 0.896312
H -4.382022 -0.049145 1.116782
H -3.290297 -2.306151 1.045735

H -0.853057 -2.468115 0.738790
H -2.537286 -0.961962 -1.569002
O -2.416838 -0.812250 -2.513915
H -1.641222 -0.227629 -2.552378
H -0.230502 1.463628 -1.731876
O -0.062665 0.880905 -2.489351
H 0.608736 0.284830 -2.137703

He2Ph, II
E = -545.014845
C -2.833079 0.887179 -0.115783
C -1.419879 0.872709 -0.120284
C -0.729507 -0.365075 -0.145314
C -1.495087 -1.553289 -0.165476
C -2.874906 -1.518308 -0.160328
C -3.554692 -0.286982 -0.135039
C 0.729507 -0.365075 -0.145314
C 1.419879 0.872709 -0.120284
C 0.677326 2.104943 -0.097415
C -0.677327 2.104942 -0.097415
C 2.833078 0.887179 -0.115783
C 3.554692 -0.286982 -0.135040
C 2.874906 -1.518308 -0.160328
C 1.495087 -1.553289 -0.165477
He 1.671261 -0.252106 3.152364
H -3.347613 1.848815 -0.095989
H -4.644259 -0.263769 -0.130678
H -3.439764 -2.450250 -0.175676
H -0.999804 -2.521818 -0.185091
H -1.231249 3.044174 -0.079455
H 1.231248 3.044174 -0.079455
H 3.347613 1.848815 -0.095990
H 4.644258 -0.263769 -0.130678
H 3.439764 -2.450250 -0.175677
H 0.999804 -2.521818 -0.185091
He -1.671255 -0.252105 3.152365

He2Ph-, II
E = -545.016328
C -2.864483 0.881843 -0.114376
C -1.430893 0.905444 -0.115787
C -0.740367 -0.362850 -0.143759
C -1.497812 -1.536968 -0.166791
C -2.900079 -1.535632 -0.163389
C -3.573609 -0.302915 -0.136938
C 0.740367 -0.362850 -0.143759
C 1.430893 0.905444 -0.115787
C 0.704731 2.108581 -0.089763
C -0.704732 2.108581 -0.089763
C 2.864483 0.881844 -0.114376
C 3.573609 -0.302914 -0.136938
C 2.900079 -1.535632 -0.163389
C 1.497812 -1.536968 -0.166791
He 1.842255 -0.289324 3.125283
H -3.395889 1.836481 -0.093400
H -4.666703 -0.278819 -0.134038
H -3.453829 -2.475040 -0.181361
H -0.990024 -2.501720 -0.187696
H -1.253646 3.052718 -0.069257
H 1.253646 3.052718 -0.069257
H 3.395889 1.836481 -0.093399
H 4.666703 -0.278819 -0.134038
H 3.453829 -2.475039 -0.181362
H 0.990024 -2.501720 -0.187697
He -1.842253 -0.289323 3.125283

He2Ph, I
E = -545.014797
C 0.000000 1.495171 -1.587275
C 0.000000 0.729468 -0.398956
C 0.000000 1.419834 0.839085
C 0.000000 2.833067 0.853698
C 0.000000 3.554821 -0.320541
C 0.000000 2.875019 -1.552159
C 0.000000 0.677309 2.071473
C -0.000000 -0.677309 2.071473
C -0.000000 -1.419834 0.839085
C -0.000000 -0.729468 -0.398956
C -0.000000 -1.495171 -1.587275
C -0.000000 -2.875019 -1.552159
C -0.000000 -3.554821 -0.320541
C -0.000000 -2.833067 0.853698
H 0.000000 -3.347597 1.815565
H 0.000000 -4.644404 -0.297194
H 0.000000 -3.439883 -2.484198
H 0.000000 -0.999983 -2.556087
H 0.000000 -1.231235 3.010827
H 0.000000 1.231235 3.010827
H 0.000000 3.347597 1.815565
H 0.000000 4.644404 -0.297194
H 0.000000 3.439883 -2.484198
H 0.000000 0.999983 -2.556087
He 3.240047 -0.000000 0.539568
He -3.240047 0.000000 0.539568

He2Ph-, I
E = -545.016353
C 0.000000 1.497835 -1.573477
C 0.000000 0.740338 -0.399200
C 0.000000 1.430818 0.869427
C 0.000000 2.864443 0.845935
C 0.000000 3.573683 -0.339055
C 0.000000 2.900138 -1.572049
C 0.000000 0.704697 2.072771
C -0.000000 -0.704697 2.072771
C -0.000000 -1.430818 0.869427
C -0.000000 -0.740338 -0.399200
C -0.000000 -1.497835 -1.573477
C -0.000000 -2.900138 -1.572049
C -0.000000 -3.573683 -0.339055

C -0.000000 -2.864443 0.845935
H -0.000000 -3.395899 1.800795
H -0.000000 -4.666795 -0.314712
H -0.000000 -3.453833 -2.511644
H -0.000000 -0.990274 -2.538497
H -0.000000 -1.253680 3.017050
H 0.000000 1.253680 3.017050
H 0.000000 3.395899 1.800795
H 0.000000 4.666795 -0.314712
H 0.000000 3.453833 -2.511644
H 0.000000 0.990274 -2.538497
He 3.244537 -0.000000 0.560448
He -3.244537 0.000000 0.560448

(H2)3Ph, II
E = -542.696046
C 3.552550 -0.269225 -0.079277
C 2.831522 0.901451 0.015969
C 1.418202 0.886872 0.018258
C 0.727603 -0.347311 -0.078875
C 1.492764 -1.532013 -0.173921
C 2.872364 -1.497063 -0.175115
C 0.676939 2.115104 0.117928
C -0.677633 2.115031 0.123203
C -1.419803 0.886967 0.024260
C -0.729953 -0.346894 -0.079964
C -2.833060 0.902069 0.024856
C -3.555160 -0.267431 -0.077813
C -2.875837 -1.494453 -0.186720
C -1.496028 -1.529979 -0.187826
H -0.317017 0.954280 -2.787420
H 0.367849 0.386543 2.730222
H 0.274344 -2.839378 2.508327
H -1.000862 -2.494618 -0.275154
H 0.996479 -2.497308 -0.244193
H 1.231217 3.051205 0.191047
H -1.231418 3.051030 0.201510
H -3.440954 -2.422359 -0.271829
H 3.346290 1.860120 0.091132
H -3.347300 1.860518 0.106918
H 3.437129 -2.426099 -0.249958
H -4.644706 -0.243861 -0.077074
H 4.642110 -0.246308 -0.080432
H 0.371293 0.739559 -3.020294
H -0.233254 0.592117 3.143072
H -0.358011 -2.464197 2.324339

(H2)3Ph-, II
E = -542.700516
C 3.602562 -0.393561 0.009405
C 2.908010 0.799527 0.003991
C 1.476334 0.839868 -0.021797
C 0.770527 -0.418576 -0.044818
C 1.513312 -1.602787 -0.035920
C 2.913570 -1.618068 -0.009947
C 0.765186 2.053566 -0.019886
C -0.644868 2.070114 -0.040547
C -1.384917 0.875261 -0.069517
C -0.709654 -0.400919 -0.079112
C -2.818057 0.869029 -0.097365
C -3.540682 -0.307013 -0.145680
C -2.881828 -1.547167 -0.163425
C -1.479955 -1.565864 -0.127381
H 0.149798 1.204293 -2.638400
H 0.478097 1.321042 2.588167
H -2.254040 -0.591214 3.261207
H -0.983421 -2.536300 -0.137394
H 0.993784 -2.561170 -0.050172
H 1.325326 2.990456 -0.001284
H -1.182445 3.020423 -0.035896
H -3.445953 -2.479525 -0.199085
H 3.450532 1.747744 0.020755
H -3.338120 1.829678 -0.083587
H 3.455718 -2.564251 -0.003372
H -4.632876 -0.269475 -0.167147
H 4.695490 -0.383057 0.029855
H 0.142091 0.912836 -3.340832
H 0.435633 1.073740 3.306440
H -2.226854 -0.635668 2.502744

(H2)3Ph, IV
E = -542.695874
C 3.552979 -0.212756 -0.185704
C 2.830436 0.955407 -0.066556
C 1.417586 0.941023 -0.077302
C 0.728123 -0.290005 -0.213140
C 1.494125 -1.471218 -0.338852
C 2.874110 -1.437003 -0.324087
C 0.674438 2.165811 0.054871
C -0.680047 2.164805 0.053145
C -1.421414 0.938925 -0.078940
C -0.730356 -0.291496 -0.211446
C -2.834321 0.951638 -0.069222
C -3.554901 -0.217983 -0.185097
C -2.874145 -1.442001 -0.316631
C -1.494329 -1.474517 -0.329444
H -1.726144 -0.456901 3.090625
H 2.169539 -0.453686 2.471259
H 0.294875 -2.668471 2.075701
H -0.997741 -2.437175 -0.426533
H 0.999359 -2.433837 -0.446062
H 1.227657 3.099924 0.157483
H -1.234726 3.098243 0.154058
H -3.438084 -2.370095 -0.407107
H 3.344370 1.911368 0.041123
H -3.349661 1.907187 0.035543
H 3.439249 -2.363785 -0.420123
H -4.644386 -0.196519 -0.173965
H 4.642413 -0.189791 -0.173327
H 1.722944 -0.443832 3.083477

H -0.172724 -3.253530 2.190693
H -2.170643 -0.432880 2.477585

(H2)3Ph-, IV
E = -542.699781
C 3.568359 -0.228429 -0.201095
C 2.856400 0.948738 -0.073632
C 1.422474 0.969371 -0.075077
C 0.735480 -0.294697 -0.207330
C 1.495666 -1.459803 -0.338915
C 2.898807 -1.454950 -0.337866
C 0.693263 2.164056 0.047890
C -0.715664 2.162109 0.042422
C -1.438171 0.962914 -0.079311
C -0.744698 -0.298138 -0.201181
C -2.871380 0.935302 -0.080656
C -3.577201 -0.246097 -0.191045
C -2.900374 -1.472288 -0.305314
C -1.499304 -1.470192 -0.306507
H -1.998956 -0.466894 3.167011
H 2.118800 -0.274257 2.415505
H 0.323108 -2.543211 2.019616
H -0.989100 -2.429815 -0.389472
H 0.990652 -2.419619 -0.448573
H 1.240015 3.104350 0.146348
H -1.266678 3.100300 0.136022
H -3.451546 -2.409560 -0.386411
H 3.385527 1.898575 0.032665
H -3.405114 1.883967 0.013177
H 3.454523 -2.387435 -0.439642
H -4.670088 -0.225174 -0.184899
H 4.661154 -0.201930 -0.193352
H 2.047386 -0.472975 3.145607
H 0.014682 -3.127540 2.393634
H -1.996301 -0.336162 2.418460

(H2)3Ph, I
E = -542.696460
C 2.833774 0.876431 -0.016114
C 1.420668 0.862203 -0.020726
C 0.730222 -0.373762 -0.085666
C 1.495166 -1.560549 -0.146018
C 2.875063 -1.526413 -0.139719
C 3.555312 -0.296669 -0.073956
C -0.727774 -0.373568 -0.084870
C -1.417614 0.862461 -0.018633
C -0.675760 2.092893 0.045000
C 0.678757 2.093056 0.043914
C -2.830589 0.877044 -0.012513
C -3.552518 -0.295984 -0.070697
C -2.872753 -1.525810 -0.138293
C -1.492872 -1.560184 -0.145539
H 2.122526 -0.491447 2.611161
H -2.150671 -0.419981 2.608197
H 0.279796 0.904893 -2.792265
H 3.348413 1.836610 0.036867
H 4.644778 -0.273845 -0.067936
H 3.439553 -2.457439 -0.185401
H 0.999826 -2.527778 -0.195568
H 1.232724 3.031036 0.092535
H -1.229638 3.030894 0.093655
H -3.345008 1.837302 0.041014
H -4.641980 -0.272837 -0.063602
H -3.437518 -2.456690 -0.183660
H -0.997677 -2.527434 -0.195603
H 1.713283 -0.336327 3.229914
H -1.725672 -0.420749 3.235719
H -0.367236 0.636905 -3.082053

(H2)3Ph-, I
E = -542.700807
C 2.863646 0.863121 0.009611
C 1.430481 0.887391 0.004944
C 0.740185 -0.376842 -0.094883
C 1.497316 -1.548358 -0.183941
C 2.898895 -1.547284 -0.179226
C 3.572482 -0.318383 -0.080209
C -0.740185 -0.376842 -0.094883
C -1.430481 0.887391 0.004943
C -0.704850 2.087779 0.096361
C 0.704850 2.087778 0.096361
C -2.863646 0.863121 0.009609
C -3.572482 -0.318383 -0.080210
C -2.898895 -1.547284 -0.179227
C -1.497316 -1.548359 -0.183941
H 1.891996 -0.526880 2.522970
H -1.891995 -0.526861 2.522974
H 0.000010 1.404601 -2.559772
H 3.394974 1.814396 0.088850
H 4.665286 -0.294911 -0.071067
H 3.452356 -2.484251 -0.247326
H 0.989412 -2.510153 -0.256997
H 1.253778 3.028850 0.169267
H -1.253779 3.028850 0.169268
H -3.394975 1.814396 0.088849
H -4.665286 -0.294911 -0.071070
H -3.452356 -2.484252 -0.247328
H -0.989411 -2.510153 -0.256997
H 1.943370 -0.594117 3.278400
H -1.943384 -0.594118 3.278401
H 0.000004 1.160438 -3.280269

(H2)3Ph, III
E = -542.695934
C -2.832633 0.843142 -0.223322
C -1.419634 0.828000 -0.231155
C -0.729307 -0.408167 -0.166514
C -1.494408 -1.594734 -0.101508
C -2.874389 -1.559505 -0.097475
C -3.554289 -0.329338 -0.157736

C 0.729305 -0.408167 -0.166515
C 1.419632 0.828000 -0.231156
C 0.677365 2.058270 -0.303516
C -0.677367 2.058270 -0.303516
C 2.832631 0.843142 -0.223325
C 3.554287 -0.329338 -0.157740
C 2.874388 -1.559505 -0.097478
C 1.494407 -1.594734 -0.101509
H -1.819935 -0.142952 3.100690
H 0.000021 2.062724 2.560730
H 1.820020 -0.142937 3.100702
H -3.347064 1.803751 -0.269973
H -4.643725 -0.305679 -0.151416
H -3.439084 -2.490095 -0.045491
H -0.999184 -2.561926 -0.049842
H -1.231180 2.996165 -0.354757
H 1.231178 2.996165 -0.354758
H 3.347062 1.803751 -0.269977
H 4.643724 -0.305679 -0.151422
H 3.439083 -2.490094 -0.045495
H 0.999183 -2.561926 -0.049843
H 2.189229 -0.474617 2.528226
H 0.000045 2.235856 3.299167
H -2.189293 -0.474525 2.528248

(H2)3Ph-, III
E = -542.700316
C -2.863753 0.848661 -0.201308
C -1.430537 0.872274 -0.207701
C -0.740225 -0.395243 -0.166014
C -1.497191 -1.569608 -0.132677
C -2.899218 -1.568212 -0.134777
C -3.572864 -0.335747 -0.168363
C 0.740224 -0.395243 -0.166014
C 1.430536 0.872274 -0.207702
C 0.704782 2.075143 -0.254352
C -0.704783 2.075143 -0.254352
C 2.863752 0.848661 -0.201310
C 3.572863 -0.335747 -0.168365
C 2.899217 -1.568213 -0.134778
C 1.497190 -1.569608 -0.132678
H -2.107636 -0.439461 3.207547
H 0.000014 1.972683 2.423716
H 2.107636 -0.439456 3.207541
H -3.395086 1.802970 -0.225509
H -4.665650 -0.311476 -0.164613
H -3.452640 -2.507205 -0.105333
H -0.989385 -2.533713 -0.100344
H -1.253755 3.018553 -0.285604
H 1.253754 3.018552 -0.285604
H 3.395085 1.802970 -0.225511
H 4.665648 -0.311477 -0.164616
H 3.452639 -2.507205 -0.105335
H 0.989384 -2.533713 -0.100344
H 2.173018 -0.562378 2.460570
H 0.000011 1.965511 3.185206
H -2.172988 -0.562365 2.460571

(H2O)3Ph, I
E = -768.432535
C 2.735691 -1.206035 0.430792
C 1.323353 -1.164506 0.429279
C 0.635997 -0.604952 -0.676524
C 1.403108 -0.114407 -1.758163
C 2.783603 -0.165828 -1.739307
C 3.460416 -0.712996 -0.632985
C -0.820125 -0.538301 -0.648546
C -1.512585 -1.027757 0.486355
C -0.775838 -1.609181 1.577796
C 0.578675 -1.676004 1.550067
C -2.921567 -0.933977 0.544520
C -3.637882 -0.373895 -0.490753
C -2.957400 0.103948 -1.626150
C -1.580744 0.020217 -1.702319
O -1.022460 2.776887 0.177821
O 0.113145 1.586801 2.458768
H 3.247751 -1.631967 1.294045
H 4.549222 -0.744798 -0.619785
H 3.350437 0.223072 -2.584616
H 0.911921 0.321704 -2.625013
H 1.128650 -2.115958 2.382386
H -1.331699 -1.994229 2.433114
H -3.436529 -1.309081 1.429446
H -4.723268 -0.300435 -0.433340
H -3.518208 0.543857 -2.450302
H -1.085817 0.401741 -2.592634
H -1.533774 2.147298 -0.339089
H -0.912848 2.352933 1.049505
H 0.070423 0.625887 2.425169
H 0.904071 1.817664 1.936762
O 1.788638 2.527060 0.407200
H 2.154567 1.847157 -0.166683
H 0.902311 2.711208 0.046355

(H2O)3Ph-, I
E = -768.455184
C 2.608891 -1.340176 -0.377743
C 1.185158 -1.267759 -0.245389
C 0.503799 -0.181533 -0.905987
C 1.259355 0.736500 -1.640348
C 2.655188 0.651586 -1.748336
C 3.320039 -0.406691 -1.104872
C -0.964956 -0.074095 -0.760761
C -1.655411 -1.060240 0.034945
C -0.941972 -2.103526 0.650150
C 0.460302 -2.202119 0.519439
C -3.074298 -0.924738 0.177693
C -3.770567 0.106581 -0.418288
C -3.097686 1.066056 -1.193859
C -1.709894 0.955345 -1.348173

O -0.685945 2.139974 1.673085
O 0.732594 -0.014769 2.848778
H 3.133257 -2.153806 0.127299
H 4.406938 -0.488873 -1.174073
H 3.209532 1.390632 -2.326164
H 0.760396 1.565225 -2.141858
H 1.000033 -3.023056 0.994887
H -1.489263 -2.833866 1.248237
H -3.603657 -1.662562 0.784186
H -4.851805 0.179365 -0.280260
H -3.640552 1.886375 -1.662971
H -1.202548 1.708989 -1.950232
H -1.132387 1.788190 0.892059
H -0.445241 1.342257 2.180925
H 0.565597 -0.747736 2.235036
H 1.449646 0.486851 2.423542
O 2.154592 1.998666 1.356104
H 2.349503 1.596889 0.499389
H 1.212935 2.243011 1.295446

He3Ph, II
E = -547.917828
C 0.348211 -0.100121 0.729456
C 0.348211 -0.100121 -0.729456
H 2.493846 -0.321948 1.000279
C -2.109356 0.152564 -0.677292
C -2.109356 0.152564 0.677292
H 2.493846 -0.321948 -1.000279
C 1.530117 -0.222418 1.495324
C -0.883376 0.026989 -1.419751
C -0.883376 0.026989 1.419751
C 1.530117 -0.222418 -1.495324
C 1.495145 -0.217873 2.875119
C -0.897982 0.029095 -2.832981
C -0.897982 0.029095 2.832981
C 1.495145 -0.217873 -2.875119
C 0.270009 -0.091027 3.554806
C 0.270009 -0.091027 -3.554806
H -3.043780 0.248402 -1.231222
H -3.043780 0.248402 1.231222
H 2.422193 -0.313580 3.440044
H -1.854827 0.127859 -3.347362
H -1.854827 0.127859 3.347362
H 2.422193 -0.313580 -3.440044
H 0.246758 -0.088082 4.644389
H 0.246758 -0.088082 -4.644389
He 2.597130 2.921020 -0.000000
He -0.445967 3.169839 0.000000
He -0.931965 -3.206758 0.000000

He3Ph-, II
E = -547.919534
C 0.347287 -0.101299 0.740315
C 0.347287 -0.101299 -0.740315
H 2.474764 -0.325435 0.990413
C -2.111303 0.156170 -0.704685
C -2.111303 0.156170 0.704685
H 2.474764 -0.325435 -0.990413
C 1.515012 -0.224550 1.497908
C -0.914481 0.031246 -1.430761
C -0.914481 0.031246 1.430761
C 1.515012 -0.224550 -1.497908
C 1.513609 -0.223870 2.900148
C -0.891086 0.029272 -2.864392
C -0.891086 0.029272 2.864392
C 1.513609 -0.223870 -2.900148
C 0.287368 -0.094554 3.573629
C 0.287368 -0.094554 -3.573629
H -3.050425 0.254535 -1.253651
H -3.050425 0.254535 1.253651
H 2.447973 -0.322473 3.453848
H -1.840719 0.129192 -3.395779
H -1.840719 0.129192 3.395779
H 2.447973 -0.322473 -3.453848
H 0.263156 -0.091713 4.666740
H 0.263156 -0.091713 -4.666740
He 2.609769 2.945038 0.000000
He -0.428459 3.192538 -0.000000
He -0.954504 -3.216163 -0.000000

He3Ph, IV
E = -547.917717
C -0.755534 -0.322252 -0.213094
C 0.703362 -0.312095 -0.234501
H -1.014081 -2.472876 -0.397355
C 0.637216 2.148432 -0.015385
C -0.717238 2.139036 0.003942
H 0.986229 -2.458836 -0.428714
C -1.514578 -1.511276 -0.304913
C 1.386641 0.926011 -0.135523
C -1.452717 0.906269 -0.093999
C 1.475787 -1.490330 -0.351116
C -2.894380 -1.485965 -0.279225
C 2.799551 0.950597 -0.156261
C -2.865761 0.910994 -0.069863
C 2.855186 -1.445462 -0.369330
C -3.580819 -0.263839 -0.160447
C 3.527772 -0.213875 -0.271412
H 1.185752 3.088001 0.059466
H -1.276366 3.070853 0.095184
H -3.453998 -2.418375 -0.351463
H 3.308394 1.912357 -0.079175
H -3.385484 1.865563 0.022745
H 3.425305 -2.369828 -0.460291
H -4.670362 -0.248224 -0.140403
H 4.617039 -0.182877 -0.286381
He -0.042225 -2.609419 2.771086
He 2.150577 -0.466002 2.944052
He -0.783028 0.373809 3.221433

He3Ph-, IV
E = -547.919342
C -0.771569 -0.325185 -0.209689
C 0.708939 -0.314671 -0.236316
H -1.009577 -2.458616 -0.384989
C 0.659341 2.148252 -0.026197
C -0.749828 2.138337 -0.002237
H 0.970825 -2.444401 -0.421776
C -1.522420 -1.500685 -0.294547
C 1.392181 0.954453 -0.141620
C -1.469014 0.934151 -0.090861
C 1.472927 -1.479208 -0.349946
C -2.924350 -1.509342 -0.268398
C 2.825615 0.941443 -0.171881
C -2.902183 0.900343 -0.065877
C 2.874946 -1.467562 -0.376514
C -3.604591 -0.285532 -0.151989
C 3.541263 -0.234083 -0.286014
H 1.202890 3.093167 0.042990
H -1.303935 3.075308 0.087651
H -3.472691 -2.449564 -0.337135
H 3.351481 1.896774 -0.101703
H -3.438865 1.847971 0.024369
H 3.433808 -2.399733 -0.466603
H -4.697617 -0.269143 -0.129308
H 4.633970 -0.201802 -0.305461
He -0.068065 -2.543061 2.851344
He 2.249220 -0.496814 2.956572
He -0.610075 0.492756 3.204323

He3Ph, I
E = -547.917934
C -1.495071 -1.559660 -0.155399
C -0.729465 -0.373254 -0.088001
C -1.419815 0.862682 -0.016397
C -2.832979 0.877129 -0.013566
C -3.554660 -0.295269 -0.079461
C -2.874888 -1.524758 -0.151181
C -0.677316 2.093085 0.053885
C 0.677315 2.093085 0.053885
C 1.419815 0.862682 -0.016397
C 0.729465 -0.373254 -0.088001
C 1.495071 -1.559660 -0.155399
C 2.874888 -1.524757 -0.151181
C 3.554660 -0.295268 -0.079461
C 2.832979 0.877130 -0.013566
H 3.347449 1.837362 0.042512
H 4.644224 -0.272099 -0.076087
H 3.439750 -2.455335 -0.203471
H 0.999877 -2.526803 -0.211497
H 1.231222 3.030946 0.107351
H -1.231223 3.030946 0.107351
H -3.347449 1.837361 0.042512
H -4.644225 -0.272100 -0.076087
H -3.439749 -2.455336 -0.203471
H -0.999877 -2.526803 -0.211496
He 0.000002 0.759303 -3.251860
He 1.821991 -0.426560 3.146884
He -1.821987 -0.426552 3.146887

He3Ph-, I
E = -547.919799
C -1.497756 -1.545288 -0.155710
C -0.740313 -0.372881 -0.088967
C -1.430827 0.893512 -0.015037
C -2.864388 0.869949 -0.015269
C -3.573538 -0.313108 -0.081750
C -2.899998 -1.544051 -0.152999
C -0.704725 2.094884 0.054759
C 0.704725 2.094884 0.054757
C 1.430827 0.893512 -0.015039
C 0.740313 -0.372881 -0.088967
C 1.497756 -1.545288 -0.155706
C 2.899998 -1.544052 -0.152996
C 3.573538 -0.313108 -0.081751
C 2.864388 0.869949 -0.015272
H 3.395758 1.823204 0.040344
H 4.666626 -0.289092 -0.078622
H 3.453729 -2.482131 -0.205761
H 0.990033 -2.508674 -0.211596
H 1.253624 3.037641 0.109285
H -1.253623 3.037641 0.109288
H -3.395758 1.823204 0.040349
H -4.666626 -0.289092 -0.078620
H -3.453729 -2.482130 -0.205766
H -0.990034 -2.508673 -0.211602
He -0.000001 0.769181 -3.268164
He 1.859409 -0.424111 3.172182
He -1.859410 -0.424116 3.172179

He3Ph, III
E = -547.917584
C 1.495217 -1.595248 -0.100657
C 0.729545 -0.409156 -0.173893
C 1.419856 0.826597 -0.250491
C 2.833066 0.841193 -0.250005
C 3.554710 -0.330823 -0.177289
C 2.875018 -1.560123 -0.102415
C 0.677277 2.056526 -0.328967
C -0.677277 2.056526 -0.328967
C -1.419856 0.826597 -0.250491
C -0.729545 -0.409156 -0.173893
C -1.495216 -1.595248 -0.100657
C -2.875018 -1.560123 -0.102416
C -3.554710 -0.330823 -0.177289
C -2.833066 0.841193 -0.250006
He -2.027313 -0.213074 2.999087
He -0.000002 2.017104 3.265710
He 2.027311 -0.213073 2.999087
H -3.347521 1.801253 -0.309065

H -4.644268 -0.307454 -0.177779
H -3.439920 -2.490380 -0.045024
H -0.999983 -2.562172 -0.041332
H -1.231228 2.993992 -0.388379
H 1.231228 2.993992 -0.388379
H 3.347521 1.801253 -0.309065
H 4.644268 -0.307454 -0.177779
H 3.439920 -2.490380 -0.045023
H 0.999983 -2.562172 -0.041332

He3Ph-, III
E = -547.919366
C 1.497931 -1.573233 -0.126190
C 0.740402 -0.400132 -0.180146
C 1.430869 0.867162 -0.240210
C 2.864514 0.843538 -0.238890
C 3.573626 -0.340171 -0.184383
C 2.900158 -1.571814 -0.127159
C 0.704669 2.069117 -0.298501
C -0.704668 2.069117 -0.298501
C -1.430868 0.867162 -0.240210
C -0.740402 -0.400132 -0.180147
C -1.497930 -1.573233 -0.126191
C -2.900157 -1.571814 -0.127160
C -3.573626 -0.340171 -0.184383
C -2.864514 0.843538 -0.238890
He -2.153374 -0.301435 3.036772
He -0.000003 1.789912 3.277565
He 2.153370 -0.301433 3.036772
H -3.395917 1.797361 -0.283566
H -4.666709 -0.316017 -0.185689
H -3.453916 -2.510376 -0.083936
H -0.990138 -2.537150 -0.081145
H -1.253621 3.012327 -0.343889
H 1.253622 3.012327 -0.343889
H 3.395917 1.797361 -0.283566
H 4.666710 -0.316017 -0.185689
H 3.453916 -2.510376 -0.083934
H 0.990138 -2.537150 -0.081143

Ph
E = -539.208102
C 0.000000 1.495331 -1.564439
C 0.000000 0.729599 -0.376023
C 0.000000 1.420006 0.862131
C 0.000000 2.833294 0.876680
C 0.000000 3.554941 -0.297637
C 0.000000 2.875198 -1.529262
C 0.000000 0.677325 2.094599
C -0.000000 -0.677325 2.094599
C -0.000000 -1.420006 0.862131
C -0.000000 -0.729599 -0.376023
C -0.000000 -1.495331 -1.564439
C -0.000000 -2.875198 -1.529262
C -0.000000 -3.554941 -0.297637
C -0.000000 -2.833294 0.876680
H -0.000000 -3.347845 1.838537
H -0.000000 -4.644526 -0.274307
H -0.000000 -3.440099 -2.461321
H -0.000000 -1.000056 -2.533208
H -0.000000 -1.231246 3.034006
H 0.000000 1.231246 3.034006
H 0.000000 3.347845 1.838537
H 0.000000 4.644526 -0.274307
H 0.000000 3.440099 -2.461321
H 0.000000 1.000056 -2.533208

Ph-
E = -539.209593
C 0.000000 1.497981 -1.549739
C 0.000000 0.740450 -0.375398
C 0.000000 1.430956 0.893382
C 0.000000 2.864633 0.869796
C 0.000000 3.573821 -0.315252
C 0.000000 2.900344 -1.548256
C 0.000000 0.704713 2.096757
C -0.000000 -0.704713 2.096757
C -0.000000 -1.430956 0.893382
C -0.000000 -0.740450 -0.375398
C -0.000000 -1.497981 -1.549739
C -0.000000 -2.900344 -1.548256
C -0.000000 -3.573821 -0.315252
C -0.000000 -2.864633 0.869796
H -0.000000 -3.396101 1.824661
H -0.000000 -4.666944 -0.290900
H -0.000000 -3.454085 -2.487882
H -0.000000 -0.990331 -2.514722
H -0.000000 -1.253682 3.041109
H 0.000000 1.253682 3.041109
H 0.000000 3.396101 1.824661
H 0.000000 4.666944 -0.290900
H 0.000000 3.454085 -2.487882
H 0.000000 0.990331 -2.514722
